# Recent Advances in Mitochondrial Fission/Fusion-Targeted Therapy in Doxorubicin-Induced Cardiotoxicity

**DOI:** 10.3390/pharmaceutics15041182

**Published:** 2023-04-07

**Authors:** Chayodom Maneechote, Siriporn C. Chattipakorn, Nipon Chattipakorn

**Affiliations:** 1 Cardiac Electrophysiology Research and Training Center, Faculty of Medicine, Chiang Mai University, Chiang Mai 50200, Thailand; 2 Center of Excellence in Cardiac Electrophysiology Research, Chiang Mai University, Chiang Mai 50200, Thailand; 3 Department of Oral Biology and Diagnostic Sciences, Faculty of Dentistry, Chiang Mai University, Chiang Mai 50200, Thailand; 4 Cardiac Electrophysiology Unit, Department of Physiology, Faculty of Medicine, Chiang Mai University, Chiang Mai 50200, Thailand

**Keywords:** doxorubicin, cardiotoxicity, mitochondrial dynamics, mitochondrial fission, mitochondrial fusion

## Abstract

Doxorubicin (DOX) has been recognized as one of the most effective chemotherapies and extensively used in the clinical settings of human cancer. However, DOX-mediated cardiotoxicity is known to compromise the clinical effectiveness of chemotherapy, resulting in cardiomyopathy and heart failure. Recently, accumulation of dysfunctional mitochondria via alteration of the mitochondrial fission/fusion dynamic processes has been identified as a potential mechanism underlying DOX cardiotoxicity. DOX-induced excessive fission in conjunction with impaired fusion could severely promote mitochondrial fragmentation and cardiomyocyte death, while modulation of mitochondrial dynamic proteins using either fission inhibitors (e.g., Mdivi-1) or fusion promoters (e.g., M1) can provide cardioprotection against DOX-induced cardiotoxicity. In this review, we focus particularly on the roles of mitochondrial dynamic pathways and the current advanced therapies in mitochondrial dynamics-targeted anti-cardiotoxicity of DOX. This review summarizes all the novel insights into the development of anti-cardiotoxic effects of DOX via the targeting of mitochondrial dynamic pathways, thereby encouraging and guiding future clinical investigations to focus on the potential application of mitochondrial dynamic modulators in the setting of DOX-induced cardiotoxicity.

## 1. Introduction

In 2040, the number of cancer cases is expected to hit 28.4 million, a 47 percent increase from 2020 [1]. Due to current medical advances in early cancer diagnosis and effective cancer treatment, the number of cancer survivors is projected to reach 22.1 million by the year 2030 [2]. However, the increasing survival of cancer patients is accompanied by an increasing number of cardiovascular issues attributable to chemotherapies [3,4]. Doxorubicin (DOX), which originally belonged to the class of drugs known as anthracyclines, is a potent cytotoxic chemotherapy drug that is frequently administered in the treatment of cancer. DOX is often used to treat solid tumors and hematological malignancies in both children and adults [3]. Unfortunately, clinical use of DOX is associated with dose-dependent and cumulative cardiotoxicity, which may result in a range of cardiovascular outcomes [4]. Oxidative stress is the most frequently documented molecular mechanism associated with DOX-induced cardiotoxicity (DIC) via altering redox status at the level of biological macromolecules. DOX primarily damages mitochondria by interacting with cardiolipin, a unique phospholipid of the inner mitochondrial membrane that helps stabilize oxidative phosphorylation (OXPHOS) complexes [5]. Mechanistically, the accumulation of DOX in the mitochondria causes the uncoupling of OXPHOS complexes, resulting in a decrease in ATP synthesis and an increase in the AMP/ATP ratio, which activates AMPK-mediated cardiomyocyte injury [5]. Moreover, DOX-induced mitochondrial dysfunction disrupts intracellular calcium homeostasis and has been demonstrated to diminish mitochondrial membrane potential, open the mPTP, and increase reactive oxygen species (ROS) production, resulting in mitochondrial dysfunction and endothelial damage [6]. Since DIC has a complex pathophysiology involving several molecular pathways, including oxidative stress, autophagy, and inflammation, emerging evidence clearly suggests that mitochondrial dynamics play a crucial role in the development of DIC.

Mitochondria are crucially responsible for the regulation of the production of ROS, energy metabolism, cell death signaling, and calcium homeostasis in cardiomyocytes [7,8,9]. Mitochondria are vulnerable to damage; however, the processes involved in the dynamics of the mitochondria, including fission and fusion, are essential for the preservation of mitochondrial function. Mitochondrial dynamics, including mitochondrial fission and fusion, are regulated by guanosine triphosphatases (GTPases) in the dynamin family [7,8,9]. The proteins in the outer mitochondrial membrane, Mitofusin 1/2 (Mfn1/2), and the inner mitochondrial membrane, Optic atrophy 1 (Opa1), facilitate mitochondrial fusion, while Dynamin-related protein 1 (Drp1) binds to its receptor proteins in the outer mitochondrial membrane, including mitochondrial fission protein 1 (MTFP1), mitochondrial fission factor (MFF), and mitochondrial fission 1 protein (Fis1), to regulate mitochondrial fission [10]. The fission activity of Drp1 can be modulated by multiple posttranslational modifications, such as phosphorylation, SUMOylation, ubiquitylation, and *S*-nitrosylation [11]. Phosphorylation of Drp1 (p-Drp1) is the most studied posttranslational modification regulating mitochondrial fission, which occurs at two serine residues, including serine-616 (p-Drp1^ser616^) and serine-637 (p-Drp1^ser637^). For p-Drp1^ser616^, it has been associated with increased Drp1 activity to promote Drp1 coordinating fission and fragmentation via the mitosis inducer and cyclin B1-cyclin dependent kinase (cyclin B1-CDK1), whereas phosphorylation by calcium calmodulin-dependent kinase (CamK) links fission to intracellular calcium signaling [11]. Unlike p-Drp1^ser616^, p-Drp1^ser637^ is phosphorylated by the cAMP-dependent protein kinase A (PKA), which inhibits mitochondrial fission through impairing Drp1 GTPase activity and preventing the translocation of Drp1 to mitochondria [12]. It has been shown that dephosphorylation of serine-637 by calcineurin appears to increase Drp1 activation and recruitment to the mitochondria, thus promoting fission. As described previously, an imbalance between mitochondrial fission and fusion is a key factor in the development of DIC. Drp1-mediated mitochondrial fission and fragmentation are greatly increased in conditions of DIC, which are associated with increased oxidative stress and ROS-mediated cardiomyocyte death. In contrast, mitochondrial fusion-related proteins, including Mfn1/2 and Opa1, were found to be downregulated throughout the course of DIC [13,14,15,16]. According to the results of prior studies, DIC pathogenesis may be effectively prevented by the modulation of mitochondrial dynamics through blocking fission or stimulating fusion [16,17,18,19,20].

Currently, DIC continues to be the most extensively researched form of antitumor drug-induced toxicity. DOX can disturb the equilibrium of mitochondrial dynamics and cause mitochondrial dysfunction. Alterations in mitochondrial quality control may also play a crucial role in the initiation and progression of DIC. Potential techniques for the prevention and treatment of DIC have been developed over the last decade by targeting mitochondrial fission/fusion homeostasis [16,17,18,19,20]. In this review, we discuss the information pertaining to the roles of mitochondrial dynamics in DIC, as well as the current breakthroughs in mitochondrial fission/fusion-targeted treatment in DIC, including the molecular signaling pathways, physiological functions, and pathological significance of DIC. This review provides insights into the anti-cardiotoxic effects which occur as a result of DOX by targeting mitochondrial dynamic pathways, the proposal being that mitochondrial fission/fusion-targeted therapy could be beneficial in preventing DIC.

## 2. Molecular Insights into DOX-Induced Cardiotoxicity: Roles of Mitochondrial Dynamics Proteins from Recent In Vitro and In Vivo Reports 

As is well known, DOX’s anti-tumor effects have been linked to its intercalation into DNA, which leads to promotion of ROS production, DNA damage, and activation of apoptosis by inhibition of topoisomerase II. However, the proposed major mechanisms of DOX cardiotoxicity are associated with several pathways, including oxidative stress, inflammation, gene expression, and programmed cell death [3,4,21]. DOX appears to promote toxic damage to cardiomyocyte mitochondria via several mitochondrial enzymes (e.g., xanthine oxidase), which contribute to the production of intracellular ROS that triggers cytotoxicity. DOX also increases superoxide generation by elevating endothelial nitric oxide synthase, which facilitates intracellular hydrogen peroxide formation to induce cellular damage [4,22,23]. Activation of nuclear factor kappa B (NF-κB), a key regulator of inflammatory reactions, has been linked to DIC-associated inflammation. Cardiomyocytes and cardiac fibroblasts produce proinflammatory cytokines, such as tumor necrosis factor alpha (TNF-α), interleukin (IL)-6, and interleukin (IL)-1β, which promote remodeling of the heart during DIC. DOX was shown to enhance activation of the nucleotide binding domain-like receptor protein 3 (NLRP3) inflammasome, which in turn activates the mammalian target of the rapamycin (mTOR)/AKT signaling pathway with subsequent impaired cardiomyocyte autophagy mechanisms [23,24]. In addition to oxidative stress and inflammation, the downregulation of cardiomyocyte gene expression (e.g., α-actin, myosin light chain, myosin heavy chain, and troponin-I) has been proposed as a potential cause of DOX’s cardiotoxicity. Alterations in those contractile proteins are linked to myofibrillar loss and diminished cardiac contractile activity, resulting in abnormal diastolic function of the heart [25]. Hence, there is evidence that DOX could promote various programmed cell death pathways in cardiomyocytes, including apoptosis, necroptosis, ferroptosis, and pyroptosis, culminating in cardiotoxicity [13,14,15,16,25,26]. Despite decades of research, the molecular mechanisms of DIC remain controversial, with various contradictions between experimental and clinical data leading to the belief that the cardiotoxic mechanisms of DOX are complicated and multifaceted.

As described previously, the molecular processes generating DIC are diverse and complicated. The specific molecular signaling pathways linked to the cardiotoxic effects of DOX remain obscure, despite substantial studies over the last few decades that have identified its putative mechanisms [3,4]. Cardiomyocyte apoptosis has been proposed as the primary mechanism. Activation of death receptors, oxidative stress, calcium dysregulation, DNA damage, and mitochondrial dysfunction are the leading causes of cardiomyocyte apoptosis [26]. The disruption of mitochondrial function through interruptions of dynamic balance has been demonstrated to play a crucial role in the development of cardiovascular and metabolic pathologies, in particular associated with DIC [16,27]. To verify the roles of mitochondrial dynamics in DIC, in vitro studies with the H9c2 rat cardiomyoblast cell line were used to study the potential mechanisms used in the case of DOX. H9c2 cells treated with DOX dosages of 0.75, 1, 2, 3, and 5 μM for 12, 16, and 24 h showed a significant increase in mitochondrial fission via upregulation of the expression of the proteins Drp1, p-Drp1^ser616^, Fis1, and MFF and downregulation of p-Drp1^ser637^, whereas mitochondrial fusion was decreased via reduction of the proteins Mfn1/2 and Opa1 [17,18,19,20,26,27,28,29,30,31,32,33,34,35,36]. In addition to H9c2 cells, neonatal rat ventricular myocytes (NRVMs) cultured in vitro have been utilized to study several cardiac molecular conditions, such as DIC. A correlation was found with mitochondrial fragmentation when NRVMs were challenged with 1.72, 3, and 5 μM of DOX for 4, 8, 24, and 48 h, as indicated by the activation of Drp1 along with the inhibition of Mfn1/2 and Opa1 [37,38,39,40,41]. Similarly, excessive Drp1-mediated mitochondrial fission was also observed after co-incubation with either 250 nM or 1 μM of DOX for 24 h in a proliferating human cardiomyocyte cell line or AC16 cells [18,32]. However, a reduction in p-Drp1^ser616^ associated with mitochondrial elongation was demonstrated in a study using adult mouse cardiomyocytes (AMCMs) treated with 1 μM of DOX for 24 h, suggesting that this controversial finding might be the differences in the study models [32]. Alteration in mitochondrial dynamics via an increase in fission and decrease in fusion could be associated with the aggravation of the severity of DIC through multiple molecular signaling pathways, including mitochondrial dysfunction, impaired autophagy and mitophagy, the promotion of oxidative stress, inflammation, and programmed cardiomyocyte death, which eventually lead to a reduction in cell viability and elevated cytotoxicity [17,18,19,20,26,27,28,29,30,31,32,33,34,35,36]. These findings suggested that DOX altered the balance of mitochondrial dynamic by inhibiting mitochondrial fusion and promoting mitochondrial fission which result in DIC pathogenesis. The roles of mitochondrial dynamics in DIC from in vitro reports are summarized in Table 1.

**Figure 1:**
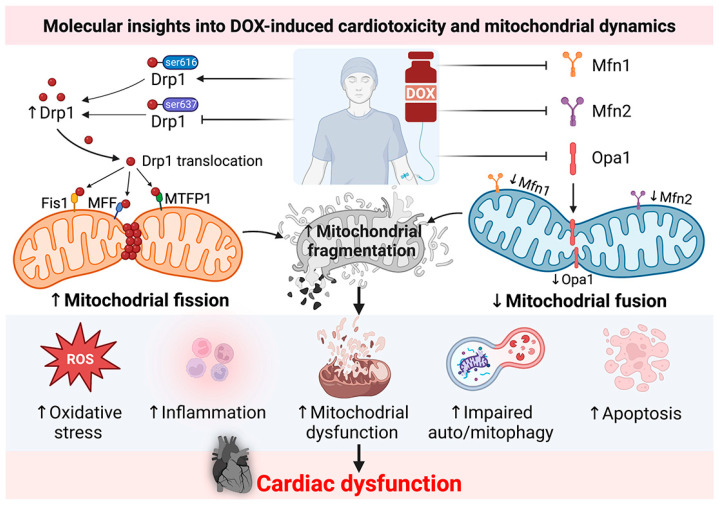
Molecular insights into the association between DOX-induced cardiotoxicity and mitochondrial dynamics pathways. DOX induces mitochondrial fission via the promotion of p-Drp1^ser616^ and inhibition of p-Drp1^ser637^ and impairs mitochondrial fusion via downregulation of Mfn1/2 and Opa1, resulting in mitochondrial dynamic imbalance, dysfunction, and fragmentation. These mechanisms could lead to increased oxidative stress, inflammation, impaired autophagy and mitophagy, and cardiomyocyte apoptosis, leading to cardiac dysfunction. Figure created with BioRender.com. DOX: doxorubicin; Drp1: dynamin-related protein 1; MFF: mitochondrial fission factor; Mfn1: mitofusin 1; Mfn2: mitofusin 2; MTFP1: mitochondrial fission process 1; Opa1: optic atrophy type 1; p-Drp1^ser616^: phosphorylation of dynamin-related protein 1 at serine 616; p-Drp1^ser637^: phosphorylation of dynamin-related protein 1 at serine 637.

Regarding the roles of mitochondrial dynamics in DIC as determined by in vivo studies, the accumulation of DOX is known to induce cardiomyocyte death via oxidative stress, inflammation, and mitochondria-dependent pathways [7,8]. The lowest accumulative dose of DOX (2.25 mg/kg) was administered in white pig models via intracoronary injection (IC). This dose of DOX was shown to upregulate the expression of fission and autophagy proteins, as well as promote fragmentation of mitochondria with severe morphological abnormalities, resulting in fibrosis and LVEF depression [44]. In mouse models, an accumulation of DOX between 12-30 mg/kg administered via intraperitoneal injection (IP) was shown to cause fragmentation of mitochondria via increased activation of mitochondrial fission, as indicated by elevation of the expression of the proteins Drp1, p-Drp1^ser616^, Fis1, and MIEF2 and a decrease of p-Drp1^ser637^ [20,28,29,36,37,39,42,45,46]. These dosages of DOX also significantly depressed mitochondrial fusion via reducing the expression of Mfn1/2 and Opa1 in those mouse models. In rat models, a wide range of DOX at 12, 12.5, 14, 15, and 18 mg/kg accumulative dosages effectively increased mitochondrial fission with impaired fusion during DIC progression [13,14,15,16,30,34,43,47,48,49]. Accordingly, administration of therapeutically effective doses could enhance fission but blunt fusion, which eventually results in fragmentation of mitochondria during progression of DIC. In addition, the precise mechanisms linking DIC to mitochondrial dynamics, specifically mitochondrial dysfunction, modification of autophagy and mitophagy, oxidative stress, inflammation, and programmed cell death have been proposed as mechanisms to provoke cardiac impairments in both morphological and functional remodeling [13,14,15,16,30,34,43,47,48,49]. Therefore, findings from in vivo models suggested that DOX induced an imbalance in mitochondrial dynamics by blocking mitochondrial fusion, encouraging mitochondrial fission, and causing DOX cardiotoxicity, as comprehensively summarized in Table 2. The molecular insights into DIC and mitochondrial dynamics are illustrated in Figure 1. 

## 3. Roles of Pharmacological Interventions Targeting Mitochondrial Fission/Fusion Therapy in DOX-Induced Cardiotoxicity: A Report from Recent In Vitro and In Vivo Studies

Up to 90 percent of the ATP necessary for sufficient cardiac contractility is produced by mitochondria in cardiomyocytes, and mitochondrial dynamics plays a key role in cardiac homeostasis [53,54,55]. DOX causes cardiomyocyte damage by modifying the structure and function of mitochondria, which is coupled with a change in mitochondrial homeostasis and dysregulation of mitochondrial fission/fusion. DOX promotes excessive generation of ROS in mitochondria and impairs mitochondrial morphology and function, resulting in the death of cardiac cells [13,14,15,16,30,34,43,47,48,49]. In addition, it has been shown that DOX induces mitochondrial fragmentation through the elevation of p-Drp1^ser616^ and reduction of Mfn1/2 and Opa1, which accelerate mitochondrial-dependent cardiomyocyte apoptotic death [13,14,15,16,30,34,43,47,48,49]. Consequently, it is necessary to confirm the targeting of mitochondrial fusion and fission proteins as potential therapeutic targets for protection against DIC by restoring the balance of mitochondrial dynamics.

As shown in Table 3 and Table 4, numerous in vitro and in vivo preclinical investigations have verified the cardioprotective impact of pharmacologically targeted mitochondrial fission/fusion treatment against DIC. Firstly, Mdivi-1 (mitochondrial division inhibitor), a putative Drp1 inhibitor, is a widely used small molecule that inhibits Drp1-dependent fission, causes the elongation of mitochondria, and reduces injury [27,56]. It has been shown that DOX treatment substantially reduced cell viability along with increased lactate dehydrogenase (LDH) levels in H9c2 cardiomyocytes, and also that the cytotoxic effects of DOX were blunted by 5.0 μM Mdivi-1 co-treatment for 48 h [16]. Moreover, increased cardiomyocyte apoptosis and p-Drp1^ser 616^ post DOX stimulation could be effectively alleviated by 1-μM Mdivi-1 pre-treatment for 30 min in H9c2 cells [28]. In the AC16 human cardiomyocyte cell line, Mdivi-1 co-treatment could prevent DOX-induced overproduction of mitochondrial superoxides and mitochondrial dysfunction by inhibiting mitophagy and preserving mitochondrial biogenesis [18]. The anti-DIC effects of Mdivi-1 have also been revealed in an in vivo study using Male Wistar rats. Co-treatment with Mdivi-1 at 1.2 mg/kg for 30 days markedly reduced DOX-induced mitochondrial dysfunction, oxidative stress, inflammation, and apoptosis, leading to improved cardiac function via modulation of mitochondrial fission/fusion proteins [16]. These results imply that suppressing fission via Mdivi-1 may be a potential therapeutic target for mitigating the cytotoxic effects of DOX.

In addition to Mdivi-1, Klotho, another anti-aging protein, has also been shown to contribute to human aging [20]. Klotho regulates energy metabolism, stress resistance, antioxidation, and calcium and mineral homeostasis. In DIC, pre-treatment with 0.1 μg/mL Klotho for 24 h significantly reduced DOX-induced p-Drp1^ser 616^-mediated apoptosis in H9c2 cardiomyocytes. As in DOX-treated mice, Klotho (0.01 mg/kg, every 48 h for 4 weeks, IP) also suppressed p-Drp1^ser616^ protein, cardiac cell death, and improved cardiac function [20]. Loulu flowers (LLF), the inflorescence of *Rhaponticum uniflorum* (L.) DC. (*R*. *uniflorum*), a member of the Compositae family, has frequently been used in treatment of various cardiovascular diseases (CVDs) [35]. Pre-treatment with LLF (200 μg/mL, 2 h) attenuated the DOX-induced aberrant expression of mitochondrial fusion/fission proteins via promotion of Opa1 and Mfn1 along with inhibition of MFF and Fis1 in H9c2 cells. LLF treatment increased cell viability while decreasing ROS production, maintaining mitochondrial membrane integrity, suppressing apoptosis, and inhibiting the DOX-induced activation of inflammation signaling in H9c2 cells [35]. 

Neuraminidase 1 (NEU1) inhibitor or oseltamivir (OSE) also protected against DIC via suppression of Drp1-dependent mitophagy [34]. NEU1, a glycosidases responsible for the removal of terminal sialic acid from glycoproteins and glycolipids, is the most abundantly expressed glycosidases in the heart, and is implicated in a number of CVDs. Pre-treatment with 10 μM OSE for 2 h could suppress Drp1-dependent mitochondrial fission and PTEN-induced kinase 1 (PINK1)/Parkin pathway-mediated mitophagy and alleviate cellular apoptosis in H9c2 cells. These cardioprotective effects of the NEU1 inhibitor against DIC were also confirmed in a rat model using OSE (20 mg/kg, 31 days, PO) [34]. Shenmai injection (SMI) is a patented traditional Chinese medicine derived from Panax ginseng and Ophiopogon japonicus, which are extensively used to treat CVDs. SMI has been shown to prevent DOX-induced excessive mitochondrial fission and insufficient mitochondrial fusion in H9c2 cardiomyocytes by increasing the ratio of L-Opa1 to S-Opa1, AMPK phosphorylation, and p-Drp1^ser637^ [33]. Furthermore, co-treatment with Liensinine, a newly discovered mitophagy inhibitor, was shown to reduce p-Drp1^ser616^ and inhibit mitochondrial fragmentation, oxidative stress, mitophagy, apoptosis, and improve mitochondrial function in cases of DOX-induced NMVMs injury [38].

Melatonin is known to influence mitochondrial homeostasis and function [30]. Pre-treatment with melatonin (10 μM, 24 h) followed by DOX exposure decreased mitochondrial fragmentation and increased ATP production, resulting in preservation of H9c2 rat cardiomyoblast viability. Similarly, in a rat model, a specific dose of melatonin (6 mg/kg) given for 14 days effectively decreased Drp1-Fis1-mediated fission and apoptosis and increased Mfn1/2-mediated fusion, cellular ATP levels, and mitochondrial biogenesis, contributing to improved cardiac function [30]. A novel angiotensin receptor, neprilysin inhibitor LCZ696, has been beneficial in treating individuals with heart failure. Pre-treatment with 20 μM LCZ696 for 30 min significantly inhibited DOX-activated Drp1 and its ser616 phosphorylation protein, thus decreasing cardiomyocyte apoptosis [28]. Vitamin D, acting as an antioxidant, was also shown to prevent DIC in a mouse in a triple-negative breast cancer model (TNBC). Pretreatment with vitamin D3 (11,500 IU/kg, 14 days) reduced p-Drp1^ser616^-associated oxidative stress and apoptosis, resulting in improved cardiac function in TNBC mice [45].

DOX is known to enhance expression of nicotinamide adenine dinucleotide phosphate (NADPH) oxidase (NOX) 1/4 and induce mitochondrial fission through activation of Drp1, leading to NLR family pyrin domain-containing 3 (NLRP3) inflammasome-mediated pyroptosis in cardiomyocytes. A specific dual NOX1/4 inhibitor, GKT137831 (60 mg/kg, given orally once a day after the first DOX injection), effectively suppressed DOX-induced Drp1-mediated mitochondrial fission and NLRP3-mediated pyroptosis in a mouse model [29]. In NRVMs cells, pre-inhibition of miR-23a using the miR-23a inhibitor (100 nM) for 48 h markedly increased viability and mitochondrial membrane potential, reduced apoptotic cell death and ROS production, inhibited expression of its target eroxisome proliferator-activated receptor gamma coactivator 1-alpha (PGC-1α), and diminished phosphorylation of Drp1 without affecting Mfn2 expression [40]. 

The managing of a balance between cardiac sympathetic and parasympathetic activity by acetylcholine receptor (AChR) agonists has been shown to be associated with mitochondrial function, cellular oxidative balance, and immunomodulation in both healthy and pathological conditions. Both an a7nAChR agonist (PNU-282987, 3 mg/kg, daily for 30 days, IP) and a mAChR agonist (bethanechol, 12 mg/kg, daily for 30 days, IP) promoted Mfn1/2-induced mitochondrial fusion and inhibited Drp1-induced mitochondrial fission, preventing DOX-induced autophagy and mitophagy [15]. In a similar manner, Luteolin (20 μM, 24 h, co-treatment) ameliorated DOX-induced toxicity in H9c2 cells via inhibition of mitochondrial fission, mitochondrial dysfunction, and apoptosis [32].

Pharmacologically induced mitochondrial fusion (M1) or hydrazone M1 has been shown to be cardioprotective in a variety of cardiovascular settings, most notably DIC. M1 dose-dependently induces mitochondrial elongation, with a requirement for basal fusion activity from Mfn1, Mfn2, or Opa1 proteins [16,27,57]. It has been strongly verified that co-treatment of DOX with M1 (2 mg/kg, daily for 30 days, IP) markedly mitigated mitochondrial dysfunction, oxidative stress, inflammation, and apoptosis, leading to improved cardiac function via modulation of mitochondrial fission/fusion proteins in a rat model. M1 (5 μM, 48h) increased cell viability while decreasing cytotoxicity in H9c2 cells following the DOX challenge [16]. 

**Figure 2:**
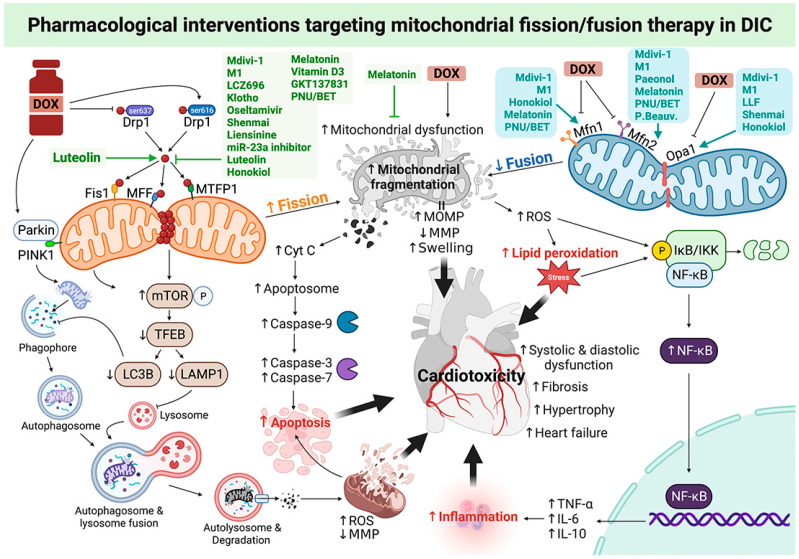
The rationale behind the pharmacological interventions targeting mitochondrial fission/fusion proteins as an effective cardioprotective strategy for DIC. Inhibiting Drp1-mediated mitochondrial fission via pharmacological fission inhibitors or promoting Mfn1/2 and Opa1-mediated mitochondrial fusion via pharmacological fusion promoters exerts cardioprotection against DIC. The underlying mechanisms of anti-DIC include prevention of DOX-induced mitochondrial fission via the promotion of p-Drp1^ser616^ and inhibition of p-Drp1^ser637^, and impaired mitochondrial fusion via downregulation of Mfn1/2 and Opa1, resulting in mitochondrial dynamic imbalance, dysfunction, and fragmentation. It also decreases ROS production, lipid peroxidation, inflammation, cardiomyocyte apoptosis, and impairments of autophagy and mitophagy, leading to reduced mitochondrial and cellular injury. Figure created with BioRender.com. Cyt c: cytochrome c; DOX: doxorubicin; Drp1: dynamin-related protein 1; IKB: nuclear factor of kappa light polypeptide gene enhancer in B-cells inhibitor; IKK: IkappaB kinase; LAMP1: lysosome-associated membrane protein-1; LC3: microtubule-associated protein 1 light chain 3; MFF: mitochondrial fission factor; Mfn1: mitofusin 1; Mfn2: mitofusin 2; MMP: mitochondrial membrane potential; mTOR: the mammalian target of rapamycin; MTFP1: mitochondrial fission process 1; Opa1: optic atrophy type 1; p-Drp1^ser616^: phosphorylation of dynamin-related protein 1 at serine 616; p-Drp1^ser637^: phosphorylation of dynamin-related protein 1 at serine 637; PINK1: PTEN-induced putative kinase 1; ROS: reactive oxygen species; TEFB: transcription elongation factor complex b.

Paeonol (Pae) is a natural antioxidant made from the root bark of Paeonia suffruticosa. It has been approved by the FDA in China to treat diseases that cause inflammation and pain [43]. It has been demonstrated that Pae inhibits ischemia-induced cardiomyocyte apoptosis via suppressing ROS, and has the ability to protect against DIC. Pae effectively enhanced Mfn2-mediated mitochondrial fusion by activating the transcription factor Stat3, which restored mitochondrial function and cardiac performance both in vivo (150 mg/kg/day, PO, post-treatment) and in vitro (50 μM, 24 h, co-treatment) under DOX conditions [43]. In addition, Honokiol (HKL), an activator of SIRT3, has been shown to increase the activation of SIRT3 and Mfn1/Opa1-mediated fusion preventing DOX-induced ROS production, mitochondrial damage, and cell death in rat neonatal cardiomyocytes (10 μM, 24 h, co-treatment). Treatment with HKL (0.2 mg/kg, daily, 45 days, IP) successfully blocked DIC in mice via promoting Mfn1/Opa1-mediated fusion, reducing mitochondrial DNA damage, and improving mitochondrial function, leading to amelioration of cardiac dysfunction [37]. Additionally, last but not least, flavonoids of *Selaginella tamariscina* (P.Beauv.) Spring (TFST) have been shown to prevent DIC by enhancing Mfn2-mediated fusion, leading to alleviation of mitochondrial dysfunction and endoplasmic reticulum stress by activating Mfn2/PERK in an in vivo mouse model (70–140 mg/kg, 8 days, PO) [46]. Taken together, targeting mitochondrial fission and fusion could be a novel potential strategy for cancer patients undergoing DOX-based chemotherapy. Roles of pharmacological interventions targeting mitochondrial fission/fusion therapy in DIC are illustrated in Figure 2.

**Table 3:** Roles of pharmacological interventions targeting mitochondrial fission/fusion therapy in DOX-induced cardiotoxicity: Recent reports from in vitro studies.

Study Model	DOX Dosage	Intervention	Major Findings					Interpretation	Ref.
			Mitochondrial Dynamics/Morphology	Mitochondrial Function/Mitophagy/Autophagy	Cell Death/Inflammation/Oxidative Stress	Cell Viability/Cytotoxicity	Cardiomyocyte Function		
H9c2 cells	0.3125 μM (48 h)	Mdivi-1 (5 μM, 48 h, co-treatment)	-	-	-	↑Cell viability ↓LDH	-	Mdivi-1 increased cell viability with decreased cytotoxicity in H9c2 cells.	[16]
H9c2 cells	5 μM (24 h)	Mdivi-1 (1 μM, 30 min, pre-treatment)	↓p-Drp1^ser616^	-	↓Anexin5-positive cells ↓c-Caspase3	-	-	Pre-treatment with Mdivi-1 and LCZ696 significantly inhibited mitochondrial fission, thus decreasing H9c2 cardiomyocyte apoptosis.	[28]
		LCZ696 (20 μM, 30 min, pre-treatment)							
AC16 cells	250 nM (24 h)	Mdivi-1 (24 h, co-treatment)	-	↓ROS ↓MMP depolarization ↑CS ↑VDAC ↓LC3II-I ↓Beclin-1 ↑p62 ↓PINK1 ↓Parkin ↑PGC1-α ↑NRF1 ↑TFAM	-	↑Cell viability	-	Mdivi-1 prevented DOX-induced mitochondrial superoxide overproduction and mitochondrial dysfunction by inhibiting mitophagy and preserving mitochondrial biogenesis.	[18]
H9c2 cells	5 μM (24 h)	Klotho (0.1 μg/mL, 24 h, pre-treatment)	↓p-Drp1^ser616^	↓MMP depolarization	↓c-Caspase3 ↓TUNEL-positive cells ↓Annexin V- positive cells	-	↑Number of cardiomyocytes ↑Peak shortening ↑+dp/dt ↑-dp/dt	Klotho pre-treatment alleviated DOX-induced cardiomyocyte dysfunction via inhibition of apoptosis, mitochondrial dysfunction, and fission in H9c2 cells.	[20]
H9c2 cells	0.75 μmol/mL (24 h)	Loulu flowers (200 μg/mL, 2 h, pre-treatment)	↑Opa1 ↑Mfn1 ↓MFF ↓Fis1	↓ROS ↓MMP depolarization	↑SOD ↓p-p65 ↓p-IKK ↓p-IkBa ↓TUNEL-positive cells ↓Bax ↑Bcl-2 ↓c-Caspase3 ↓c-Caspase9 ↓c-PARP	-	-	Pre-treatment with LLF attenuated the DOX-induced decrease in viability, ROS production, apoptosis, inflammation, and aberrant expression of mitochondrial dynamics-related proteins in H9c2 cells.	[35]
H9c2 cells	1 μM (12 h)	NEU1 inhibitor oseltamivir (10 μM, 2 h, pre-treatment)	↓Drp1	↓ATG5 ↓Beclin-1 ↓LC3I/II ↓PINK1 ↓Parkin ↓p62	↓NEU1 ↓c-Caspase3 ↓c-Caspase9 ↓TUNEL-positive cells	-	-	Pre-treatment with a NEU1 inhibitor could suppress Drp1-dependent mitochondrial fission and PINK1/Parkin pathway-mediated mitophagy and alleviate cellular apoptosis in H9c2 cells.	[34]
H9c2 cells	1 μM (16 h)	Shenmai injection (0.5%, 8 h, pre-treatment)	↑Aspect ratio ↑Form factor ↑L-Opa1/S-Opa1 ↑p-Drp1^ser637^ ↑p-AMPK^Thr172^	↓ROS ↓MMP depolarization ↓OCR	↓Bax/Bcl-2 ↓c-Caspase3 ↓Annexin V-positive cells	↑Cell viability	-	Shenmai rescued DOX-injured H9c2 cardiomyocytes from apoptosis and mitochondrial dysfunction by preventing DOX-induced excessive mitochondrial fission and insufficient mitochondrial fusion.	[33]
NMVMs	5 μM (24 h)	Liensinine (20 μM, 24 h, co-treatment)	↓Fragmented mitochondria ↓p-Drp1^ser616^	↓MMP depolarization ↓ROS ↑TOM20 ↑TIM23 ↓Rab7 ↓LRRK2	↓TUNEL-positive cells ↓p-ERK	-	-	Liensinine suppressed Drp1-mediated mitochondrial fragmentation, mitochondrial dysfunction, and apoptosis in NMVM cardiomyocytes.	[38]
H9c2 cells	3 μM (24 h)	Melatonin (10 μM, 24 h, pre-treatment)	↓Fragmented mitochondria	↑ATP	-	↑Cell viability	-	Pre-treatment with melatonin followed by DOX exposure decreased mitochondrial fragmentation and increased ATP production, resulting in preserved H9c2 rat cardiomyoblast viability.	[30]
NRVMs	3 μM (24 h)	miR-23a inhibitor (100 nM, 48 h, pre-treatment)	↔Mfn2 ↓p-Drp1	↓MMP depolarization ↓ROS ↑PGC1-α	↓miR-23a ↓Dead cells ↓Cyt c ↓c-Caspase3	↑Cell viability	-	Inhibition of miR-23a attenuated NRVMs cardiomyocyte damage by directly targeting PGC-1α/p-Drp1, thereby inhibiting mitochondria-dependent apoptosis.	[40]
H9c2 cells	1 μM (24 h)	Luteolin (20 μM, 24 h, co-treatment)	↓Fragmented mitochondria ↓p-Drp1^ser616^	↓ROS	↑SOD activity ↓Apoptotic cells ↓Bax ↑Bcl-2 ↑Bcl-xL ↓c-Caspase3	-	-	Luteolin ameliorated DOX-induced toxicity in H9c2 cells via inhibiting mitochondrial fission, mitochondrial dysfunction, and apoptosis.	[32]
H9c2 cells	0.3125 μM (48 h)	M1 (5 μM, 48 h)	-	-	-	↑Cell viability ↓LDH	-	M1 increased cell viability with decreased cytotoxicity in H9c2 cells.	[16]
Primary cardiomyocytes	3 μM (24 h)	Paeonol (50 μM, 24 h, co-treatment)	↓Mitochondrial number ↑Mitochondrial size ↑Mfn2	↓MMP depolarization ↓ROS ↑OCR	↓Apoptotic cells	↑Cell viability ↓LDH		Pae enhanced Mfn2-mediated mitochondrial fusion, restored mitochondrial function and cardiomyocyte viability under the DOX conditions.	[43]
NRVMs	5 μM (24 h)	Honokiol (10 μM, 24 h, co-treatment)	↓Fragmented mitochondria ↑Opa1 ↑Mfn1 ↓Drp1	↓MnSOD	↓Apoptotic cells	-	-	Honokiol increased the activation of SIRT3 and Mfn1/Opa1-mediated fusion to prevent DOX-induced ROS production, mitochondrial damage, and cell death in rat neonatal cardiomyocytes.	[37]
AMCM cells	1 μM (24 h)	Luteolin (10 μM, 24 h, co-treatment)	↑p-Drp1^ser616^ ↓Mitochondrial elongation	↓ROS ↓MMP depolarization ↑LAMP1 ↑TFEB ↓p-mTOR^ser2448^	↓TUNEL-positive cells ↓Bax ↓c-Caspase9 ↑Bcl2 ↑LC3BII ↑PINK1 ↑Parkin ↑Bnip3	↓LDH ↓CK	↑Cell length ↑-dL/dt ↑+dL/dt	Luteolin reduced DOX-induced ROS accumulation, MMP collapse, and apoptosis by facilitating autophagosome formation and improving lysosomal function, possibly through a Drp1/mTOR/TFEB-dependent mechanism.	[19]

Abbreviations: AMPK: AMP-activated protein kinase; ATG5: autophagy-related gene 5; ATP: adenosine triphosphate; Bax: Bcl-2-associated X protein; Bcl-2: B-cell lymphoma-2; CK: creatine kinase; CMDL-1: cardiomyocyte mitochondrial dynamic-related lncRNA 1; COM: complex protein; Cyt c: cytochrome c; DOX: doxorubicin; Drp1: dynamin-related protein 1; ECAR: extracellular acidification rates; Fis1: mitochondrial fission 1 protein; FoxO1: forkhead box protein O1; IKBα: nuclear factor of kappa light polypeptide gene enhancer in B-cells inhibitor, alpha; IKK: IkappaB kinase; LAMP1: lysosome-associated membrane protein-1; LC3: microtubule-associated protein 1 light chain 3; LDH: lactate dehydrogenase; LRRK2: mutations in the gene encoding for leucine-rich repeat kinase 2; MFF: mitochondrial fission factor; Mfn1: mitofusin 1; Mfn2: mitofusin 2; MIEF2: mitochondrial elongation factor 2; MMP: mitochondrial membrane potential; mTOR: the mammalian target of rapamycin; NRF1: nuclear respiratory factor 1; OCR: oxygen consumption rate; Opa1: optic atrophy type 1; p-Drp1ser616: phosphorylation of dynamin-related protein 1 at serine 616; p-Drp1 ser637: phosphorylation of dynamin-related protein 1 at serine 637; PARP: poly (ADP-ribose) polymerase; PGC1-α: peroxisome proliferator-activated receptor-gamma coactivator-1 alpha; PINK1: PTEN induced putative kinase 1; ROS: reactive oxygen species; SOD: superoxide dismutase; TEFB: transcription elongation factor complex b; TFAM: transcription factor A, mitochondrial; TUNEL: terminal deoxynucleotidyl transferase dUTP nick end labeling.

**Table 4:** Roles of pharmacological interventions targeting mitochondrial fission/fusion therapy in DOX-induced cardiotoxicity: recent reports from in vivo studies.

Study Model	DOX Dosage	Intervention	Major Findings				Interpretation	Ref.
			Mitochondrial Dynamics/Morphology	Mitochondrial Function/Mitophagy/Autophagy/	Oxidative Stress/Inflammation/Cell Death	Heart Parameters		
Male Wistar rats	18 mg/kg (3 mg/kg, 6 doses, days 0, 4, 8, 15, 22, and 29, IP)	Mdivi-1 (1.2 mg/kg, daily for 30 days, IP)	↓Drp1 ↓p-Drp1^ser616^ ↑Mfn1 ↑Mfn2 ↑Opa1 ↓Mitochondrial volume density ↑Mitochondrial area	↑RCR ↓ROS ↓MMP depolarization ↓Swelling ↓Parkin ↓Beclin-1 ↓p62 ↓LC3II/I	↓MDA in serum and tissue ↓TNF-α ↓IL-6 ↓Bax ↓c-Caspase3 ↓Cyt c ↓TUNEL	↑HR ↑LVESP ↑+dp/dt ↑SV ↑SBP ↑DBP ↑EF ↑FS ↑E/A ratio ↓LVEDP ↓-dp/dt ↓LF/HF ratio ↓NT-proBNP ↓Troponin-I	Mdivi-1 reduced mitochondrial dysfunction, oxidative stress, inflammation, and apoptosis, leading to improved cardiac function via modulation of mitochondrial fission/fusion proteins.	[16]
C57BL/6 mice	12 mg/kg (4 mg/kg at days 0, 7, and 14, IP)	Klotho (0.01 mg/kg, every 48 h for 4 weeks, IP)	↓p-Drp1^ser616^ ↑Mitochondrial length	↑ATP content	↓c-Caspase3	↑HW/BW ↓CK activity ↓Fibrosis ↓Disorganized myofibers ↑EF ↑FS ↓LVESD ↓LVEDD	Klotho alleviated DOX-induced cardiotoxicity by reducing apoptosis and mitochondrial fission through downregulation of Drp1 expression.	[20]
Male Sprague Dawley rats	15 mg/kg (2.5 mg/kg, 6 times within 2 weeks, IP)	NEU1 inhibitor oseltamivir (20 mg/kg, 31 days, PO)	↓Drp1	↓LC3II ↓Beclin-1 ↓ATG5 ↑p62 ↓PINK1 ↓Parkin	↓NEU1 ↓LDH ↑T-AOC ↑GSH ↑SOD ↓H_2_O_2_ ↓c-Caspase3 ↓c-Caspase9 ↓Bax ↓Bad ↑Bcl-2 ↓TUNEL-positive cells	↑EF ↑FS ↓LVEDs ↑Cardiomyocyte area ↓Fibrosis ↑Cross sectional area ↓c-TnT ↓CKMB	A NEU1 inhibitor effectively suppressed Drp1-dependent mitochondrial fission, autophagy, mitophagy, and apoptosis, leading to attenuation of cardiac dysfunction and remodeling in a rat model.	[34]
Female Sprague Dawley rats	12 mg/kg (4 mg/kg, on days 4, 8 and 12, IP)	Melatonin (6 mg/rat/day, 14 days, drinking water)	↑Mfn1 ↑Mfn2 ↓Drp1 ↓hFis1	↓PINK1 ↑PGC1-α ↑SIRT1	↓c-Caspase3 ↓c-PARP	↑Cardiac output ↑Total work performance ↑HW	Melatonin effectively decreased Drp1-Fis1-mediated fission and apoptosis, increased Mfn1/2-mediated fusion, cellular ATP levels, and mitochondrial biogenesis, contributing to improved cardiac function in a rat model.	[30]
Female Balb/c mice	30 mg/kg (10 mg/kg, on days 2, 8 and 15, IP)	Vitamin D3 (11,500 IU/kg, 14 days, pre-treatment)	↓p-Drp1^ser616^	-	↓4-HNE ↓NQO1 ↓c-Caspase3	↑EF ↑SV ↑FS	Vitamin D3 pretreatment reduced mitochondrial fission-associated oxidative stress and apoptosis, resulting in improved cardiac function in a mouse model.	[45]
C57BL/6J mice	12 mg/kg (4 mg/kg, 3 weekly injections at 0, 7, and 14 days, IP)	GKT137831 (dual inhibitor of NOX1 and NOX4) (60 mg/kg, once a day after the first DOX injection, PO)	↓p-Drp1^ser616^ ↑p-Drp1^ser637^ ↑Mitochondrial length/width	-	↓NLRP3 ↓c-Caspase1 ↓IL1-b ↓IL1-18 ↓p-NF-kB ↓NOX1 ↓NOX4 ↓GSDMD-NT	↑EF ↑FS ↓LVESD ↓LVEDD ↑HW/BW	GKT137831 effectively suppressed DOX-induced Drp1-mediated mitochondrial fission and the consequent NLRP3 inflammasome activation and pyroptosis in a mouse model.	[29]
Male Wistar rats	18 mg/kg (3 mg/kg, 6 doses, days 0, 4, 8, 15, 22, and 29, IP)	a7nAChR agonist (PNU: PNU-282987, 3 mg/kg, daily for 30 days, IP)	↓Drp1 in mitochondria ↓p-Drp1^ser616^ ↑Mfn1 ↑Mfn2	↑RCR ↓ROS ↓MMP depolarization ↓Swelling ↓PINK1 ↓Parkin ↓Beclin-1 ↓p62 ↓LC3II/I	↓MDA in serum and tissue ↓TNF-α ↓IL-6 ↓Bax ↓c-Caspase3 ↓TUNEL-positive cells	↑HR ↑SV ↑SBP ↑DBP ↑EF ↑FS ↑E/A ratio ↓LF/HF ratio ↓NT-proBNP ↓Troponin-I	a7nAChR agonists and mAChR agonists effectively protected the heart against DOX cardiotoxicity via promoting Mfn1/2-induced mitochondrial fusion and inhibiting Drp1-induced mitochondrial fission, as well as reducing mitochondrial dysfunction, autophagy, mitophagy, inflammation, and apoptosis in rats.	[15]
		mAChR agonist (BET: bethanechol, 12 mg/kg, daily for 30 days, IP)						
Male Wistar rats	18 mg/kg (3 mg/kg, 6 doses, days 0, 4, 8, 15, 22, and 29, IP)	M1 (2 mg/kg, daily for 30 days, IP)	↓Drp1 ↓p-Drp1^ser616^ ↑Mfn1 ↑Mfn2 ↑Opa1 ↓Mitochondrial volume density ↑Mitochondrial area	↑RCR ↓ROS ↓MMP depolarization ↓Swelling ↓Parkin ↓Parkin ↓Beclin-1 ↓p62 ↓LC3II/I	↓MDA in serum and tissue ↓TNF-α ↓IL-6 ↓Bax ↓c-Caspase3 ↓Cyt c ↓TUNEL	↑HR ↑LVESP ↑+dp/dt ↑SV ↑SBP ↑DBP ↑EF ↑FS ↑E/A ratio ↓LVEDP ↓−dp/dt ↓LF/HF ratio ↓NT-proBNP ↓Troponin-I	M1 reduced mitochondrial dysfunction, oxidative stress, inflammation, and apoptosis, leading to improved cardiac function via modulation of mitochondrial fission/fusion proteins.	[16]
Male Sprague Dawley rats	15 mg/kg (5 mg/kg, on days 1, 6 and 11, 3 times/2 weeks, IP)	Paeonol (150 mg/kg, daily for 3 days, PO, post-treatment)	↑Mitochondrial size ↑Mfn2	↑Complex-III	↓LDH ↓CK-MB	↑EF ↑FS ↓LVESD ↑LVSP ↓LVEDP ↑+dp/dt ↑−dp/dt	Pae enhanced Mfn2-mediated mitochondrial fusion, restored mitochondrial function and cardiac performance in DIC rats.	[43]
Male BALB/c mice	15 mg/kg (5 mg/kg, every 15 days for a total of three doses, IP)	Honokiol (0.2 mg/kg, daily, 45 days, IP)	↓Fragmented mitochondria ↓Mitochondrial damage ↑Opa1 ↑Mfn1	↑Sirt3 ↑OGG1 ↓MnSOD ↓8-Oxo-dG ↓Mitochondrial citrate synthase ↑ATP	↓TUNEL-positive cells ↑Bcl-2	↓HW/TL ↑FS ↓Fibrosis	Honokiol successfully blocked DIC in mice via promotion of Mfn1/Opa1-mediated fusion, reducing mitochondrial DNA damage, and improving mitochondrial function, leading to amelioration of cardiac dysfunction.	[37]
Male C57BL/6 mice	20 mg/kg (5 mg/kg, at first, third, fifth and seventh day, IP)	Total flavonoids of *Selaginella tamariscina* (P.Beauv.) Spring (70–140 mg/kg, 8 days, PO)	↑Mfn2	↑PPAR-α ↑PGC1-α ↑Sirt3 ↓PERK ↓ATF4 ↓CHOP	↓LDH ↑SOD ↓MDA ↑GSH ↑CAT ↓Bcl-2 ↓Bax ↓Caspase9 ↓Cyt c	↑EF ↑FS ↑E/A ratio ↓CKMB ↓BNP ↓cTnT ↓Fibrosis	Total flavonoids of *Selaginella tamariscina* effectively prevented DIC by enhancing Mfn2-mediated fusion, leading to alleviation of mitochondrial dysfunction and endoplasmic reticulum stress by activating Mfn2/PERK in a mouse model.	[46]

Abbreviations: 4HNE: 4-Hydroxynonenal, or 4-hydroxy-2-nonenal; a7nAChR: alpha-7 nicotinic receptor; Akt: protein kinase B; ATG5: autophagy-related gene 5; ATP: adenosine triphosphate; Bax: Bcl-2-associated X protein; BNP: brain natriuretic peptide; BW: body weight; c-TnT: cardiac troponin T; CAT: catalase; CK: creatine kinase; CKMB: creatine kinase MB; CO: cardiac output; CS: mitochondrial citrate synthase; Cx43: connexin 43; CypD1: cyclophilin D; Cyt c: cytochrome c; Drp1: dynamin-related protein 1; E/A ratio: the ratio between E-wave and A-wave; EF: ejection fraction; Erk: extracellular signal-regulated kinase; Fis1: mitochondrial fission 1 protein; FoxO1: forkhead box O1; Foxo3: forkhead box O3; FS: fractional shortening; GSDMD-NT: gasdermin D-N terminal; GSH: glutathione; H2O2: hydrogen peroxide; HO-1: heme oxygenase 1; HR: heart rate; HW: heart weight; IC: intracoronary injection; IL: interleukin; IP: intraperitoneal injection; Keap1: Kelch-like ECH-associated protein 1; LC3: microtubule-associated protein 1 light chain 3; LDH: lactate dehydrogenase; LF/HF ratio: low-frequency to high-frequency ratio; LVDP: left ventricular developed pressure; LVEDD: left ventricular end-diastolic diameter; LVEF: left ventricular ejection fraction; LVESD: left ventricle end-systolic dimension; M2AchR: muscarinic acetylcholine receptor M2; MDA: malondialdehyde; Mfn1: mitofusin 1; Mfn2: mitofusin 2; MIEF2: mitochondrial elongation factor 2; MMP: mitochondrial membrane potential; NFkB: nuclear factor kappa-light-chain-enhancer of activated B cells; NLRP3: NLR family pyrin domain-containing 3; NOX: NADPH oxidases; NQO1: NAD(P)H:quinone oxidoreductase 1; Nrf1: nuclear respiratory factor 1; Nrf2: nuclear respiratory factor 2; NT-proBNP: N-terminal pro B-type natriuretic peptide; OGG1: 8-oxoguanine DNA glycosylase-1; Opa1: optic atrophy type 1; OXPPHOS: oxidative phosphorylation; p-Drp1ser616: phosphorylation of dynamin-related protein 1 at serine 616; p-Drp1 ser637: phosphorylation of dynamin-related protein 1 at serine 637; p-PDH: pyruvate dehydrogenase; PARP: Poly (ADP-ribose) polymerase; PGC1-α: peroxisome proliferator-activated receptor gamma coactivator 1-alpha; RCR: respiratory control ratio; ROS: reactive oxygen species; SIRT1: sirtuin 1; Sirt3: sirtuin 3; SOD: superoxide dismutase; TLR-4: toll-like receptor 4; TNF-α: tumor necrosis factor alpha.

## 4. Roles of Non-Pharmacological Interventions Targeting Mitochondrial Fission/Fusion Therapy in DOX-Induced Cardiotoxicity: A Report from Recent In Vitro and In Vivo Studies

In addition to pharmacological interventions, the roles of non-pharmacological therapies targeting mitochondrial fission and fusion in DOX cardiotoxicity have been reported. Both in vitro and in vivo research findings regarding those non-pharmacological interventions are summarized in Table 5 and Table 6. Primarily, Drp1 knockdown has been shown to prevent Drp1-dependent mitochondrial fragmentation, mitophagy flux, and H9c2 cell death. Drp1-deficient mice were consistently rescued from DOX-induced mitochondrial fragmentation, mitochondrial degradation by the lysosomes, and myocardial injury, providing strong evidence for a role for Drp1-associated mitochondrial fragmentation in DIC [36]. 

It has been claimed that Foxo3a inhibits DOX-induced mitochondrial fission and apoptosis in cardiomyocytes. This is due to Foxo3a being downregulated in cardiomyocytes and in the mouse heart in response to DOX administration [42]. Cardiac specific Foxo3a transgenic mice showed a reduction in mitochondrial dynamics protein of 49 kDa (MIEF2)-mediated mitochondrial fission, apoptosis, and cardiotoxicity upon DOX exposure. Knockdown of MIEF2 reduced DOX-induced mitochondrial fission and apoptosis in cardiomyocytes and in vivo. Additionally, knockdown of MIEF2 protected the heart from DOX-induced cardiotoxicity [42]. Additionally, cardiomyocyte mitochondrial dynamic-related lncRNA 1 (CMDL-1) is markedly downregulated in cardiomyocytes by the DOX challenge, which targets the Drp1 protein. Lentiviral overexpression of CMDL-1 for 24 h prevented DOX-induced p-Drp1^ser637^-mediated mitochondrial fission and apoptosis in H9c2 cardiomyocytes [31]. Likewise, loss of Rubicon, an inhibitory interacting partner of autophagy protein UVRAG, ameliorated DOX-induced cardiotoxicity through enhancement of Opa1-mediated mitochondrial fusion and the improvement of autophagic flux and mitophagy in Rubicon-deficient mice [52]. 

Heme oxygenase-1 (HO-1) is a transcriptional stress response gene that is increased in pathological conditions of the heart and other organ systems [50]. Mice that overexpressed human HO-1 were rescued from DOX-induced dilated cardiomyopathy, cardiac remodeling, and infiltration of CD11b. Cardiac-specific HO-1 overexpression reduced DOX-mediated dilatation of the sarcoplasmic reticulum and mitochondrial disorganization, including a reduction in both mitochondrial fragmentation and increased numbers of damaged mitochondria in autophagic vacuoles. Overexpression of HO-1 increased NRF1, PGC1-α, and TFAM protein expression, which accelerated mitochondrial biogenesis. HO-1 overexpression also reduced Fis1 upregulation and enhanced Mfn1 and Mfn2 expression. PINK1 and Parkin, mitophagy pathway mediators, were similarly prevented from altering mitochondrial dynamics [50]. Moreover, early implantation of mitochondria (Mito) or exogenous mitochondrial administration into the left myocardium could effectively protect the heart against DOX-induced dilated cardiomyopathy in rats. Mito (500 μg/rat intramyocardial injection) effectively inhibited Drp1-mediated fission and promoted Mfn2-mediated fusion, reducing oxidative stress, autophagy, apoptosis, and mitochondrial damage, leading to preservation of LVEF and myocardial remodeling in DIC rats [48]. 

In addition, an increase in Mfn2 levels in NRVMs cardiomyocytes via a CRISPR activation plasmid (48 h, pre-treatment) attenuated the DOX-induced increase in mitochondrial fission and prevented mitochondrial ROS production, thereby preventing DOX-induced apoptosis of cardiomyocytes [41]. In DOX-treated cardiomyocytes, restoration of Mfn2-mediated mitochondrial fusion with adenoviruses encoding the Mfn2 gene (Ad-Mfn2) (48 h, pre-treatment) increased mitochondrial oxidative metabolism, decreased cellular injury and apoptosis, and inhibited mitochondria-derived oxidative stress. Transgenic mice with cardiac-specific Mfn2 exhibited preserved mitochondrial fusion and diminished myocardial damage when treated with DOX [39]. Targeting Mfn2-mediated mitochondrial fusion may therefore provide a double therapeutic benefit in DOX-based chemotherapy by simultaneously preventing DIC.

**Figure 3:**
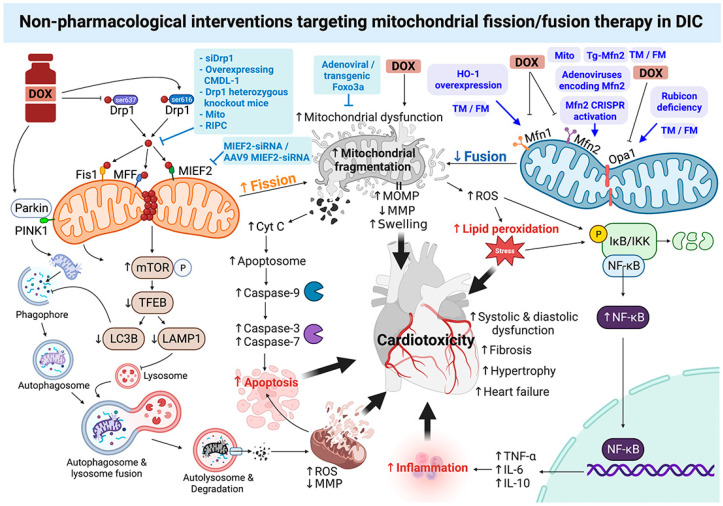
The rationale behind the non-pharmacological interventions targeting mitochondrial fission/fusion proteins as an effective cardioprotective strategy for DIC. Non-pharmacological inhibition of fission or promotion of fusion provides anti-DIC via prevention of DOX-induced p-Drp1^ser616^ activation and p-Drp1^ser637^ dephosphorylation and Mfn1/2 and Opa1 downregulation, resulting in suppression of mitochondrial dynamic imbalance, dysfunction, and fragmentation. Furthermore, it reduces ROS production, lipid peroxidation, inflammation, cardiomyocyte apoptosis, and impairments of autophagy and mitophagy, which reduce mitochondrial and cellular damage. Figure created with BioRender.com. Cyt c: cytochrome c; DOX: doxorubicin; Drp1: dynamin-related protein 1; IKB: nuclear factor of kappa light polypeptide gene enhancer in B-cells inhibitor; IKK: IkappaB kinase; LAMP1: lysosome-associated membrane protein-1; LC3: microtubule-associated protein 1 light chain 3; MFF: mitochondrial fission factor; Mfn1: mitofusin 1; Mfn2: mitofusin 2; MMP: mitochondrial membrane potential; mTOR: the mammalian target of rapamycin; MTFP1: mitochondrial fission process 1; Opa1: optic atrophy type 1; p-Drp1ser616: phosphorylation of dynamin-related protein 1 at serine 616; p-Drp1 ser637: phosphorylation of dynamin-related protein 1 at serine 637; PINK1: PTEN induced putative kinase 1; ROS: reactive oxygen species; TEFB: transcription elongation factor complex b.

In pig models, remote ischemic conditioning (RIPC) applied immediately before each DOX injection resulted in preservation of cardiac contractility with significantly higher long-term left ventricular ejection fraction (LVEF) and less cardiac fibrosis through prevention of mitochondrial fragmentation and dysregulated autophagy [44]. Endurance treadmill training (TM) and voluntary free wheel activity (FW) also prevented DOX-induced mPTP opening and apoptosis, mitochondrial dynamic alterations, and increases in autophagy and mitophagy signaling [49]. In summary, inhibition of mitochondrial fission and promotion of fusion hold potential in translating a targeted mitochondrial therapy for DIC into a new clinical setting. The roles of non-pharmacological interventions targeting mitochondrial fission/fusion therapy in DIC are illustrated in Figure 3.

**Table 5:** Roles of non-pharmacological interventions targeting mitochondrial fission/fusion therapy in DOX-induced cardiotoxicity: Recent reports from in vitro studies.

Study Model	DOX Dosage	Intervention	Major Findings				Interpretation	Ref.
			Mitochondrial Dynamics/Morphology	Mitochondrial Function/Mitophagy/Autophagy	Cell Death/Inflammation/Oxidative Stress	Cell Viability/Cytotoxicity		
H9c2 cells	750 nM (24 h)	siDrp1 (knockdown of Drp1)	↑Mitochondrial size ↑Form factor ↑Aspect ratio ↑Mitochondrial number	↓Mitophagy foci	↓PI-positive cells ↓c-PARP ↓c-Caspase3	-	Drp1 knockdown decreased mitochondrial fragmentation, mitophagy flux, and apoptosis in H9c2 cardiomyocytes.	[36]
Mice cardiomyocytes	3 μM (24h)	Adenoviral Foxo3a (24h, pre-treatment)	↓Fragmented mitochondria	-	↑Foxo3a ↓TUNEL-positive cells ↓c-Caspase3	-	Promoting Foxo3a and inhibiting MIEF2 attenuates DOX-induced mitochondrial fission and apoptosis in cardiomyocytes.	[42]
		Adenoviral MIEF2-siRNA (24h, pre-treatment)	↓Fragmented mitochondria ↓MIEF2	-	↓TUNEL-positive cells ↓c-Caspase3	-		
H9c2 cells	2 μM (24h)	Lentiviral overexpressing CMDL-1 (24h, pre-treatment)	↑CMDL-1 ↓Fragmented mitochondria ↓Drp1 ↑p-Drp1^ser637^	-	↓Apoptotic cells	-	CMDL-1 played an anti-apoptotic role in DOX cardiotoxicity by promoting p-Drp1^ser637^-mediated mitochondrial fission and fragmentation.	[31]
NRVMs	1.72 μM (4, 8, 24, and 48 h)	Mfn2 CRISPR activation plasmid (48 h, pre-treatment)	↑Mfn2 (48 h) ↓Fragmented mitochondria (4 h)	↓ROS (8 h)	↓Caspase3 activity (8 h) ↓TUNEL-positive cells (24 h)	-	An increase in cardiomyocyte levels of Mfn2 attenuated the DOX-induced increase in mitochondrial fission and prevented mitochondrial ROS production, thus leading to the prevention of the DOX-induced cardiomyocyte apoptosis.	[41]
NRVMs	3 μM (24 h)	Adenoviruses encoding the Mfn2 gene (Ad-Mfn2) (48 h, pre-treatment)	↑Mitochondrial size ↓Mitochondrial number ↑Mfn2	↓MMP depolarization ↓ROS ↑OCR ↑ECAR ↑CI ↑CIII	↓Caspase3 activity ↓TUNEL-positive cells ↓Cyt c ↓FoxO1	↑Cell viability ↓LDH	Restoration of Mfn2-mediated mitochondrial enhanced mitochondrial oxidative metabolism reduced cellular injury and apoptosis, and inhibited mitochondria-derived oxidative stress in the DOX-treated cardiomyocytes.	[39]

Abbreviations: AMPK: AMP-activated protein kinase; ATG5: autophagy-related gene 5; ATP: adenosine triphosphate; Bax: Bcl-2-associated X protein; Bcl-2: B-cell lymphoma-2; CK: creatine kinase; CMDL-1: cardiomyocyte mitochondrial dynamic-related lncRNA 1; COM: complex protein; Cyt c: cytochrome c; DOX: doxorubicin; Drp1: dynamin-related protein 1; ECAR: extracellular acidification rates; Fis1: mitochondrial fission 1 protein; FoxO1: forkhead box protein O1; IKBα: nuclear factor of kappa light polypeptide gene enhancer in B-cells inhibitor, alpha; IKK: IkappaB kinase; LAMP1: lysosome-associated membrane protein-1; LC3: microtubule-associated protein 1 light chain 3; LDH: lactate dehydrogenase; LRRK2: mutations in the gene encoding for leucine-rich repeat kinase 2; MFF: mitochondrial fission factor; Mfn1: mitofusin 1; Mfn2: mitofusin 2; MIEF2: mitochondrial elongation factor 2; MMP: mitochondrial membrane potential; mTOR: the mammalian target of rapamycin; NRF1: nuclear respiratory factor 1; OCR: oxygen consumption rate; Opa1: optic atrophy type 1; p-Drp1ser616: phosphorylation of dynamin-related protein 1 at serine 616; p-Drp1 ser637: phosphorylation of dynamin-related protein 1 at serine 637; PARP: poly (ADP-ribose) polymerase; PGC1-α: peroxisome proliferator-activated receptor-gamma coactivator-1 alpha; PINK1: PTEN induced putative kinase 1; ROS: reactive oxygen species; SOD: superoxide dismutase; TEFB: transcription elongation factor complex b; TFAM: transcription factor A, mitochondrial; TUNEL: Terminal deoxynucleotidyl transferase dUTP nick end labeling.

**Table 6:** Roles of non-pharmacological interventions targeting mitochondrial fission/fusion therapy in DOX-induced cardiotoxicity: Recent reports from in vivo studies.

Study Model	DOX Dosage	Intervention	Major Findings				Interpretation	Ref.
			Mitochondrial Dynamics/Morphology	Mitochondrial Function/Mitophagy/Autophagy	Oxidative Stress/Inflammation/Cell Death	Heart Parameters		
Mice	15 mg/kg (single dose, euthanized after 3 days, IP)	The Drp1 heterozygous knockout mice (Drp1^+/−^)	↑Mitochondrial length ↑Mitochondrial width ↑Mitochondrial size	↓LC3-II	↓LDH ↓c-Caspase3 ↓Oxyblot ↓4HNE ↓p-PDH	↓cTnI	Drp1-deficient mice were protected from DOX-induced cardiac damage via inhibiting Drp1-dependent mitochondrial fragmentation, autophagy, and apoptosis.	[36]
Male C57BL/6 mice	20 mg/kg (5 mg/kg, weekly for 4 consecutive weeks, IP)	Foxo3a transgenic model (pre-treatment)	↓Fragmented mitochondria	-	↓TUNEL-positive cells	↓LVIDd ↑FS	Foxo3a overexpression and knockdown of MIEF2 attenuated DOX-induced mitochondrial fission and apoptosis, leading to improved cardiac function in a mouse model.	[42]
		AAV9 MIEF2-siRNA (2 × 1011 vector genomes (vg)/mouse, pre-treatment)						
FVB/NJ mice (Rubicon-deficient mice)	20 mg/kg (single dose, euthanized on day 3, IP)	Rubicon-deficiency-generated by piggyBac transposition	↓Fragmented mitochondria ↑Opa1	↓Rubicon ↑ATP ↓LC3II ↓P62 ↑Parkin	↓LDH ↓ROS	↓Fibrosis ↓CK-MB	Loss of Rubicon ameliorated DOX-induced cardiotoxicity through enhancement of Opa1-mediated mitochondrial fusion and improvement of autophagic flux and mitophagy in Rubicon-deficient mice.	[52]
Male and female mixed FVB/C57BL/6 mice	18 mg/kg (6 mg/kg, every third day for a week, 14 days, IV)	Humanized HO-1 overexpressing (HBAC)	-	-	↑HO-1 ↓CD11b^+^	↑EF ↓EDD ↓ESD ↓LVEDD ↓LVESD ↑LVPWtd ↑LVPWts	Mice globally overexpressing human HO-1 were protected from DOX-induced dilated cardiomyopathy, cardiac cytoarchitectural derangement, and infiltration of mononuclear phagocytes. Cardiac-specific overexpression of HO-1 ameliorated DOX-mediated dilation of the sarcoplasmic reticulum as well as mitochondrial fragmentation and increased numbers of damaged mitochondria in autophagic vacuoles.	[50]
		Cardiac-specific HO-1 overexpression (cs-HO-1)	↓Mitochondrial number ↑Mitochondrial area ↑Mfn1 ↑Mfn2 ↓Fis1	↑mtDNA ↑Pol-grammar ↑COX3 ↑Nrf1 ↑PGC1-α ↑TFAM ↑PINK1	-	-		
Adult male Sprague Dawley rats	12.5 mg/kg (3.125 mg/kg, 4 separated time points, once every 5 days, 20 days, IP)	Implantation of mitochondria (Mito) (500 μg/rat, day-21 after DOX induction, euthanized by day 60, intramyocardial injection)	↓Drp1 ↑Mfn2	↑PGC1-α ↑TFAM ↑Nrf1 ↑Nrf2 ↑EER-a ↑mitoDNA expression ↑Beclin-1 ↑ATG-5 ↑LC3-II/I ↓CypD1 ↑OXPHOS	↓γ-H2AX-positive cells ↓NOX1 ↓NOX2 ↓p22phox ↓Oxidative index ↓c-Caspase3 ↓Bax ↓c-PARP ↓Cyt c	↑EF ↓HW/Tibial length ↓LW/Tibial length ↓Fibrosis ↓Hypertrophy ↑Cx43 ↓BNP+	Implantation of mitochondria effectively inhibited Drp1-mediated fission and promoted Mfn2-mediated fusion, reducing oxidative stress, autophagy, apoptosis, and mitochondrial damage, leading to preservation of LVEF and myocardial remodeling in DIC rats.	[48]
Male C57BL/6 mice	15 mg/kg (5 mg/kg, consecutive 3 weeks, IP)	Cardiac-specific Mfn2 Transgenic mice (Tg-Mfn2)	↑Mfn2 ↓Mitochondrial number ↑Mitochondrial size	↓ROS	↓TUNEL-positive cells ↓Caspase3 activity ↓MDA ↑SOD ↓FoxO1	↑EF ↓LVESV ↓cTnT ↓Fibrosis	Restoration of Mfn2-mediated mitochondrial fusion reduced cardiac dysfunction, cellular injury/apoptosis, and mitochondria-derived oxidative stress in DOX-treated mice.	[39]
White pigs	2.25 mg/kg (0.45 mg/kg, weeks 0, 2, 4, 6, and 8, IC)	RIPC (3 cycles of 5 min leg ischemia followed by 5 min reperfusion)	↓Mitochondrial fragmentation ↓Drp1	↓Beclin-1 ↓p62 ↓mitDNA content	-	↑LVEF ↑Wall thickening ↓Fibrosis	RIPC applied immediately before each DOX injection resulted in preservation of cardiac contractility with significantly higher long-term LVEF and less cardiac fibrosis through prevention of mitochondrial fragmentation and dysregulated autophagy in a pig model.	[44]
Male Sprague Dawley rats	14 mg/kg (2 mg/kg, 7 weekly injections, IP)	Treadmill exercise (TM) (velocity from 18 to 27 m/min, 60 min/day, 5 days/week, 12 weeks, co-treatment)	↑Mfn1 ↑Mfn2 ↑Opa1 ↔Drp1	↓Swelling ↓Beclin-1 ↓LC3-II ↔p62 ↓PINK1	↓Caspase3 ↓Caspase8 ↓Caspase9 ↓Bax/Bcl-2	-	Endurance treadmill training and voluntary freewheel activity prevented DOX-increased mitochondrial dysfunction and apoptotic signaling, alterations in mitochondrial dynamics, and increases in autophagy and mitophagy signaling in a rat model.	[49]
		Running wheel exercise (FW) (24 h/day, 12 weeks, co-treatment)						
Male C57BL/6J mice	20 mg/kg (5 mg/kg, weekly for 4 weeks, IP)	A motorized treadmill (speed of 13–15 m/min for 60 min/d for 4 wk, post-treatment)	↑Drp1 ↓Mfn2 ↓Opa1	↑LC3II ↓p62 ↓ULK1^ser757^ ↓p-mTOR ↑PGC1-α ↓NRF1 ↓COXII ↓COXIII	↓c-Caspase3 ↓TUNEL-positive cells ↓Protein carbonyl ↓4HNE ↓IL-1b ↓p22^phox^ ↓p67^phox^ ↓p47^phox^	↓Abnormal morphology	A motorized treadmill exercise prevented DOX-induced apoptosis and mitigated tissue damage via increased mitophagy flux, increased Drp1-mitochondrial fission, and decreased fusion markers (Opa1 and Mfn2) in a mouse model.	[51]

Abbreviations: 4HNE: 4-hydroxynonenal, or 4-hydroxy-2-nonenal; a7nAChR: alpha-7 nicotinic receptor; Akt: protein kinase B; ATG5: autophagy-related gene 5; ATP: adenosine triphosphate; Bax: Bcl-2-associated X protein; BNP: brain natriuretic peptide; BW: body weight; c-TnT: cardiac troponin T; CAT: catalase; CK: creatine kinase; CKMB: creatine kinase MB; CO: cardiac output; CS: mitochondrial citrate synthase; Cx43: connexin 43; CypD1: cyclophilin D; Cyt c: cytochrome c; Drp1: dynamin-related protein 1; E/A ratio: the ratio between E-wave and A-wave; EF: ejection fraction; Erk: extracellular signal-regulated kinase; Fis1: mitochondrial fission 1 protein; FoxO1: forkhead box O1; Foxo3: forkhead box O3; FS: fractional shortening; GSDMD-NT: gasdermin D-N terminal; GSH: glutathione; H2O2: hydrogen peroxide; HO-1: heme oxygenase 1; HR: heart rate; HW: heart weight; IC: intracoronary injection; IL: interleukin; IP: intraperitoneal injection; Keap1: Kelch-like ECH-associated protein 1; LC3: microtubule-associated protein 1 light chain 3; LDH: lactate dehydrogenase; LF/HF ratio: low-frequency to high-frequency ratio; LVDP: left ventricular developed pressure; LVEDD: left ventricular end-diastolic diameter; LVEF: left ventricular ejection fraction; LVESD: left ventricle end-systolic dimension; M2AchR: muscarinic acetylcholine receptor M2; MDA: malondialdehyde; Mfn1: mitofusin 1; Mfn2: mitofusin 2; MIEF2: mitochondrial elongation factor 2; MMP: mitochondrial membrane potential; NFkB: nuclear factor kappa-light-chain-enhancer of activated B cells; NLRP3: NLR family pyrin domain-containing 3; NOX: NADPH oxidases; NQO1: NAD(P)H: quinone oxidoreductase 1; Nrf1: nuclear respiratory factor 1; Nrf2: nuclear respiratory factor 2; NT-proBNP: N-terminal pro B-type natriuretic peptide; OGG1: 8-oxoguanine DNA glycosylase-1; Opa1: optic atrophy type 1; OXPPHOS: oxidative phosphorylation; p-Drp1ser616: phosphorylation of dynamin-related protein 1 at serine 616; p-Drp1 ser637: phosphorylation of dynamin-related protein 1 at serine 637; p-PDH: pyruvate dehydrogenase; PARP: poly (ADP-ribose) polymerase; PGC1-α: peroxisome proliferator-activated receptor gamma coactivator 1-alpha; RCR: respiratory control ratio; ROS: reactive oxygen species; SIRT1: sirtuin 1; Sirt3: sirtuin 3; SOD: superoxide dismutase; TLR-4: Toll-like receptor 4; TNF-α: tumor necrosis factor alpha.

## 5. Existing Controversial Reports on Mitochondrial Fission/Fusion-Targeted Therapy in DOX-Induced Cardiotoxicity

As mentioned above, inhibiting fission via downregulation of the Drp1 protein or promoting fusion via upregulation of the Mfn1/2 and Opa1 proteins has been shown to be anti-DIC in both in vitro and in vivo experiments. However, there are some conflicting findings regarding mitochondrial fission/fusion-targeted therapy in DIC. It has been demonstrated that luteolin, a natural product that is extracted from vegetables and fruits, possesses properties that are anti-oxidative, anti-tumorigenic, and anti-inflammatory [32]. Luteolin application in adult mouse cardiomyocytes or AMCM cells at a concentration of 10 M for a period of 24 h as a co-treatment overtly alleviated DOX-induced cardiomyocyte contractile dysfunction, inhibited apoptosis, accumulation of ROS, and loss of mitochondrial membrane potential via promotion of mitochondrial autophagy in association with facilitating p-Drp1^ser616^, with reduced mitochondrial elongation, and upregulation of transcription factor EB (TFEB) expression [32] (Table 3). Likewise, motorized treadmill exercise (speed of 13–15 m/min for 60 min per day for 4 weeks after treatment) prevented DOX-induced apoptosis and mitigated tissue damage via an increase in mitophagy flux, an increase in Drp1-mitochondrial fission, and a decrease in fusion markers (Opa1 and Mfn2) [51] (Table 6). Although the major findings from these two studies demonstrated that promoting fission via upregulation of the Drp1 protein or inhibiting fusion via downregulation of the Mfn2 and Opa1 proteins exhibited anti-DIC efficacy, further studies are still required to validate the protective role of the promotion of Drp1-mediated fission and the inhibition of Mfn1/Mfn2/Opa1-mediated fusion against DIC conditions.

## 6. Conclusions

Although DOX is one of the most widely used chemotherapeutic treatments effective in the increase in the survival of cancer patients, DIC remains a key limiting factor in the use of this drug. Therapies that target the mitochondrial dynamic pathways have appeared as possible preventative and therapeutic options for DIC. Although mitochondrial fission/fusion-targeted remedies could be cardioprotective regimens to protect against DOX’s life threatening cardiotoxic effects, limited clinical evidence is available. Future clinical investigations are needed to warrant the use of these mitochondrial dynamic-targeted interventions in a clinical setting.

## Figures and Tables

**Table 1:** Roles of mitochondrial dynamics in DOX-induced cardiotoxicity: Recent reports from in vitro studies.

Study Model	DOX Dosage	Major Findings				Interpretation	Ref.
		Mitochondrial Dynamics/Morphology	Mitochondrial Function/Mitophagy/Autophagy	Oxidative Stress/Inflammation/ Cell Death	Cell Viability/Cytotoxicity/Function		
AC16 cells	250 nM (24 h)	↑Drp1	↑ROS ↑MMP depolarization ↓mtDNA ↓PGC1-α ↓NRF1 ↓TFAM ↑PINK1 ↑Parkin ↑LC3II-I ↑Beclin-1 ↓p62	-	↓Cell viability	DOX induced Drp1-mediated mitochondrial fission, leading to toxic effects in mitochondria and cytotoxicity in cardiomyocyte cell lines.	[18]
H9c2 cells	750 nM (24 h)	↓Opa1 ↓Mfn2 ↓Mitochondrial size ↓Form factor ↓Aspect ratio ↓Mitochondrial number	↑Mitophagy foci	↑PI-positive cells ↑c-PARP ↑c-Caspase3	-	DOX impaired mitochondrial fusion and fragmentation, resulting in impaired mitophagy and apoptosis in cardiomyocytes.	[36]
H9c2 cells	0.75 μM (24 h)	↑MFF ↑Fis1 ↓Mfn1 ↓Opa1	↑ROS ↑MMP depolarization	↓SOD ↑p-p65 ↑p-IKK ↓p-IkBα ↑TUNEL-positive cells ↑Bax ↓Bcl-2 ↑c-Caspase3 ↑c-Caspase9 ↑c-PARP	↓Cell viability	DOX suppressed mitochondrial fusion and promoted fission, causing mitochondrial dysfunction, inflammation, and apoptosis, in addition to reduced cardiomyocyte viability.	[35]
H9C2 cells	1 μM (12 h)	↑Drp1	↑PINK1 ↑Parkin ↑ATG5 ↑Beclin-1 ↑LC3I/II ↓p62	↑NEU1 ↑c-Caspase3 ↑c-Caspase9 ↑TUNEL-positive cells	-	DOX increased mitochondrial fission, mitophagy, and autophagy, contributing to cardiomyocyte apoptosis.	[34]
H9c2 cells	1 μM (16 h)	↑p-Drp1^ser616^ ↓p-Drp1^ser637^ ↓L-Opa1/S-Opa1 ↓Aspect ratio ↓Form factor	↑ROS ↑MMP depolarization ↓OCR ↓p-AMPK^Thr172^	↑Bax/Bcl-2 ↑c-Caspase3 ↑Annexin V-positive cells	↓Cell viability	DOX injured cardiomyocytes via increasing mitochondrial fragmentation, mitochondrial dysfunction, and apoptosis.	[33]
AMCM cells	1 μM (24 h)	↓p-Drp1^ser616^ ↑Mitochondrial elongation	↑ROS ↑MMP depolarization ↓LAMP1 ↓TFEB ↑p-mTOR^ser2448^ ↓LC3B-II ↓PINK1 ↓Parkin ↓Bnip3	↑TUNEL-positive cells ↑Bax ↑c-Caspase9 ↓Bcl-2	↑LDH ↑CK ↓Cell length ↓−dL/dt ↓+dL/dt	DOX induced cardiomyocyte contractile dysfunction and apoptosis via impairing mitochondrial dynamics and dysfunction, mitophagy, and autophagy.	[19]
H9c2 cells/AC16 cells	1 μM (24 h)	↑p-Drp1^ser616^ ↑Fragmented mitochondria	↑ROS	↓SOD activity ↑Apoptotic cells ↑Bax ↓Bcl-2 ↓Bcl-xL ↑c-Caspase3	-	DOX-mediated mitochondrial fission and fragmentation contribute to mitochondrial dysfunction and cardiomyocyte apoptosis.	[32]
NRVMs	1.72 μM (4, 8, 24, and 48 h)	↓Mfn2 (8/24 h) ↑Fragmented mitochondria (4 h)	↑ROS (8 h)	↑Caspase3 activity (8 h) ↑TUNEL-positive cells (24 h)	-	DOX reduced mitochondrial fusion, leading to mitochondrial fragmentation, dysfunction, and cardiomyocyte apoptosis.	[41]
H9c2 cells	2 μM (24 h)	↑Drp1 ↓p-Drp1^ser637^ ↓CMDL-1 ↑Fragmented mitochondria	-	↑Apoptotic cells	-	DOX promoted mitochondrial fission and fragmentation, resulting in cardiomyocyte apoptosis.	[31]
Mouse cardiomyocytes	3 μM (24 h)	↑Fragmented mitochondria ↑MIEF2	-	↓Foxo3a ↑TUNEL-positive cells ↑c-Caspase3	-	Foxo3a is downregulated in the cardiomyocytes in response to DOX treatment, leading to increased mitochondrial fission and apoptosis.	[42]
NRVMs	3 μM (24 h)	↑p-Drp1 ↓Mfn2	↑MMP depolarization ↑ROS ↓PGC1-α	↑miR-23a ↑Cyt c ↑c-Caspase3	↓Cell viability	DOX increased mitochondrial fission while decreasing fusion, resulting in mitochondrial dysfunction, miR-23a upregulation, and cardiomyocyte apoptosis.	[40]
H9c2 cells	3 μM (24 h)	↑Fragmented mitochondria	↓ATP	-	↓Cell viability	DOX reduced cardiomyocyte viability via mitochondrial fragmentation and dysfunction.	[30]
NRVMs	3 μM (24 h)	↓Mfn2 ↓Mitochondrial size ↑Mitochondrial number	↑MMP depolarization ↑ROS ↓OCR ↓ECAR ↓COM-I ↓COM-III	↑FoxO1 ↑Caspase3 activity ↑TUNEL-positive cells ↑Cyt c	↓Cell viability ↑LDH	DOX increased cardiomyocyte toxicity by inhibiting mitochondrial fusion-induced fragmentation, activating mitochondrial dysfunction, and inducing FoxO1-associated apoptosis.	[39]
Primary cardiomyocytes	3 μM (24 h)	↓Mfn2 ↑Mitochondrial number ↓Mitochondrial size	↑MMP depolarization ↑ROS ↓OCR	↑Apoptotic cells	↓Cell viability ↑LDH	DOX reduced cardiomyocyte viability with increased toxicity via mitochondrial fusion-mediated fragmentation, leading to mitochondrial dysfunction and apoptosis.	[43]
H9c2 cells	5 μM (24 h)	↑p-Drp1^ser616^	↑MMP depolarization	↑c-Caspase3 ↑TUNEL-positive cells ↑Annexin V-positive cells	↓Cardiomyocyte number ↓Peak shortening ↓+dp/dt ↓−dp/dt	DOX induced cardiomyocyte dysfunction via promoting apoptosis, mitochondrial dysfunction, and fission.	[20]
NMVMs	5 μM (24 h)	↑p-Drp1^ser616^ ↑Fragmented mitochondria	↑MMP depolarization ↑ROS ↓TOM20 ↓TIM23 ↑Rab7 ↑LRRK2	↑TUNEL-positive cells ↑p-ERK	-	DOX increased cardiomyocyte apoptosis by impairing excessive mitochondrial fission, fragmentation, and dysfunction.	[38]
H9c2 cells	5 μM (24 h)	↑p-Drp1^ser616^ ↓p-Drp1^ser637^	↑ROS	↑c-Caspase1	-	DOX induced mitochondrial fission and dysfunction and cardiomyocyte apoptosis.	[29]
H9c2 cells	5 μM (24 h)	↑p-Drp1^ser616^	-	↑Anexin5-positive cells ↑c-Caspase3	-	DOX induced mitochondrial fission and cardiomyocyte apoptosis.	[28]
NRVMs	5 μM (24 h)	↑Drp1 ↓Opa1 ↓Mfn1 ↑Fragmented mitochondria	↑MnSOD	↑Apoptotic cells	-	DOX induced mitochondrial fission with impaired fusion, leading to mitochondrial dysfunction and cardiomyocyte apoptosis.	[37]

Abbreviations: AMPK: AMP-activated protein kinase; ATG5: autophagy-related gene 5; ATP: adenosine triphosphate; Bax: Bcl-2-associated X protein; Bcl-2: B-cell lymphoma-2; CK: creatine kinase; CMDL-1: cardiomyocyte mitochondrial dynamic-related lncRNA 1; COM: complex protein; Cyt c: cytochrome c; DOX: doxorubicin; Drp1: dynamin-related protein 1; ECAR: extracellular acidification rates; Fis1: mitochondrial fission 1 protein; FoxO1: forkhead box protein O1; IKBα: nuclear factor of kappa light polypeptide gene enhancer in B-cells inhibitor, alpha; IKK: IkappaB kinase; LAMP1: lysosome-associated membrane protein-1; LC3: microtubule-associated protein 1 light chain 3; LDH: lactate dehydrogenase; LRRK2: mutations in the gene encoding for leucine-rich repeat kinase 2; MFF: mitochondrial fission factor; Mfn1: mitofusin 1; Mfn2: mitofusin 2; MIEF2: mitochondrial Elongation Factor 2; MMP: mitochondrial membrane potential; mTOR: the mammalian target of rapamycin; NRF1: nuclear respiratory factor 1; OCR: oxygen consumption rate; Opa1: optic atrophy type 1; p-Drp1ser616: phosphorylation of dynamin-related protein 1 at serine 616; p-Drp1 ser637: phosphorylation of dynamin-related protein 1 at serine 637; PARP: poly (ADP-ribose) polymerase; PGC1-α: peroxisome proliferator-activated receptor-gamma coactivator-1 alpha; PINK1: PTEN induced putative kinase 1; ROS: reactive oxygen species; SOD: superoxide dismutase; TEFB: transcription elongation factor complex b; TFAM: transcription factor A, mitochondrial; TUNEL: Terminal deoxynucleotidyl transferase dUTP nick end labeling.

**Table 2:** Roles of mitochondrial dynamics in DOX-induced cardiotoxicity: Recent reports from in vivo studies.

Study Model	DOX Dosage	Major Findings				Interpretation	Ref.
		Mitochondrial Dynamics/Morphology	Mitochondrial Function/Mitophagy/Autophagy	Oxidative stress/Inflammation/Cell Death	Heart Parameters		
Male Sprague Dawley rats (Langendorff protocol)	1 μM (120 min)	↑p-Drp1	-	↑p-Akt ↑p-Erk1/2 ↑p-p53	↓LVDP ↓HR ↓Coronary flow ↓Time taken to depolarization ↓Time taken to hypercontracture	DOX caused a reduction in cardiac function by promoting mitochondrial fission and cell death.	[17]
White pigs	2.25 mg/kg (0.45 mg/kg, weeks 0, 2, 4, 6, and 8, IC)	↑Drp1 ↑Mitochondrial fragmentation	↑Beclin-1 ↑p62 ↑mitDNA content	-	↓LVEF ↓Wall thickening ↑Fibrosis	DOX induced cardiac dysfunction via upregulation of fragmented mitochondria with severe morphological abnormalities, together with upregulation of fission and autophagy proteins.	[44]
C57BL/6 mice	12 mg/kg (4 mg/kg at days 0, 7, and 14, IP)	↑p-Drp1^ser616^ ↓Mitochondrial length	↓ATP content	↑c-Caspase3	↓HW/BW ↑CK activity ↑Fibrosis ↑Disorganized myofibers ↓EF ↓FS ↑LVESD ↑LVEDD	DOX induced cardiotoxicity by promoting apoptosis and mitochondrial fission.	[20]
C57BL/6J mice	12 mg/kg (4 mg/kg, 3 weekly injections at 0, 7, and 14 days, IP)	↑p-Drp1^ser616^ ↓p-Drp1^ser637^ ↓Mitochondrial length/width	-	↑NLRP3 ↑c-Caspase1 ↑IL1-b ↑IL1-18 ↑p-NF-kB ↑NOX1 ↑NOX4 ↑GSDMD-NT	↓EF ↓FS ↑LVESD ↑LVEDD ↓HW/BW	DOX promoted mitochondrial fission and NLRP3 inflammasome hyperactivation to trigger pyroptotic cell death, which is causally linked to progressive myocardial dysfunction.	[29]
Female Sprague Dawley rats	12 mg/kg (4 mg/kg, on days 4, 8 and 12, IP)	↑Drp1 ↑hFis1 ↔Mfn1 ↓Mfn2	↑PINK1 ↑Parkin ↓PGC1-α ↓SIRT1	↑c-Caspase3 ↑c-PARP	↓CO ↓Total work performance ↓HW	DOX increased mitochondrial fission while impairing fusion and mitophagy, resulting in apoptosis as well as mitochondrial and cardiac dysfunction.	[30]
Male Sprague Dawley rats	12.5 mg/kg (3.125 mg/kg, once every 5 days, 4 separated time points within 20 days, sacrifice at day 60, IP)	↑Drp1 ↑Mfn2	↑LC3BII/I ↑OXPHOS	↑TLR-4 ↑MyD88 ↑p-NF-kb ↑IL-1B ↑TNF-α ↑NOX1 ↑NOX2 ↑Cyt c ↑c-Caspase3 ↑c-Caspase9 ↑Bax ↑Apoptotic cells	↓EF ↑Fibrosis ↑Hypertrophy	DOX caused inflammation, oxidative stress, mitochondrial dysfunction, autophagic/apoptotic signaling pathways, and LV dysfunction.	[47]
Adult male Sprague Dawley	12.5 mg/kg (3.125 mg/kg, 4 separated time points, once every 5 days, 20 days, IP)	↑Drp1 ↑Mfn2	↓mitoDNA ↑PGC1-α ↓OXPHOS ↑CypD1 ↑Nrf1 ↑Beclin-1 ↑ATG-5 ↑LC3-II/I	↑NOX1 ↑NOX2 ↑c-Caspase3 ↑Bax ↑c-PARP ↑Cyt c	↓EF ↑HW/Tibial length ↑LW/Tibial length ↑Fibrosis ↑Hypertrophy ↓Cx43 ↑BNP+	DOX induced inflammation, oxidative stress, mitochondrial damage, and impaired autophagic/apoptotic signaling pathways, all of which contributed to LV dysfunction.	[48]
Male Sprague Dawley rats	14 mg/kg (2 mg/kg, 7 weekly injections, IP)	↓Mfn1 ↓Mfn2 ↓Opa1 ↑Drp1	↑Swelling ↑Beclin-1 ↑LC3-II ↑p62 ↑PINK1	↑Caspase3 ↑Caspase8 ↑Caspase9 ↑Bax/Bcl-2	-	DOX treatment resulted in augmentation of mPTP susceptibility and apoptotic signaling, decreased expression of fusion-related proteins, increased Drp1, and the activation of autophagy and mitophagy signaling.	[49]
Male Sprague Dawley rats	15 mg/kg (2.5 mg/kg, 6 times within 2 weeks, IP)	↑Drp1	↑LC3II ↑Beclin-1 ↑ATG5 ↓p62 ↑PINK1 ↑Parkin	↑NEU1 ↑LDH ↓GSH ↓SOD ↑H_2_O_2_ ↑c-Caspase3 ↑c-Caspase9 ↑Bax ↑Bad ↓Bcl-2 ↑TUNEL-positive cells	↓EF ↓FS ↑LVEDs ↓Cardiomyocyte area ↑Fibrosis ↓Cross sectional area ↑c-TnT ↑CKMB	Elevated NEU1 triggered by DOX increased mitochondrial fission, autophagy, and mitophagy, leading to a maladaptive feedback loop towards myocardial apoptosis, cell death, and cardiac dysfunction.	[34]
Mice	15 mg/kg (single dose, euthanized after 3 days, IP)	↓Mitochondrial length ↓Mitochondrial width ↓Mitochondrial size	↑LC3-II	↑LDH ↑4HNE ↑p-PDH ↑c-Caspase3	↑c-TnI	DOX induced myocardial injury via mitochondrial fragmentation, accelerated mitophagy flux, oxidative stress, and apoptosis.	[36]
Adult male Balb/c mice	15 mg/kg (3 times/week for 2 weeks, IP)	↑Drp1 ↑Mitochondrial length/width	↓ATP	↑TUNEL-positive cells ↑c-Caspase3	↑LVEDD ↑LVESD ↓EF ↑NT-proBNP ↑Fibrosis ↑Hypertrophy	Reduced cardiac function, mitochondrial morphology disturbance, reduced activity of mitochondrial respiration complex I and lowered ATP content were detected post-DOX stimulation in mice.	[28]
Male C57BL/6 mice	15 mg/kg (5 mg/kg, 3 consecutive weeks, IP)	↓Mfn2 ↑Mitochondrial number ↓Mitochondrial size	↑ROS	↑MDA ↓SOD ↑FoxO1 ↑TUNEL-positive cells ↑Caspase3 activity	↓EF ↑LVESV ↑c-TnT ↑Fibrosis	DOX-induced upregulation of FoxO1 inhibited Mfn2-mediated mitochondrial fusion and promoted mitochondrial dysfunction and oxidative stress, resulting in cardiac dysfunction.	[39]
Male Sprague Dawley rats	15 mg/kg (5 mg/kg, on days 1, 6 and 11, 3 times/2 weeks, IP)	↓Mitochondrial size ↓Mfn2	↓Complex-III	↑LDH	↓EF ↓FS ↑LVESD ↓LVSP ↑LVEDP ↓+dp/dt ↓-dp/dt ↑CKMB	DOX induced damage by inhibiting mitochondrial fusion via the PKCe-Stat3-Mfn2 pathway, leading to cardiac dysfunction.	[43]
Male BALB/c mice	15 mg/kg (5 mg/kg, every 15 days for a total of three doses, IP)	↓Opa1 ↓Mfn1 ↑Fragmented mitochondria ↑Mitochondrial damage	↓Sirt3 ↓OGG1 ↑MnSOD ↑8-Oxo-dG ↓CS ↓ATP	↑TUNEL-positive cells ↓Bcl-2	↑HW/TL ↓FS ↑Fibrosis	DOX-induced cardiotoxicity is associated with increased ROS production and consequent fragmentation of mitochondria and cell death.	[37]
Male Wistar rats	18 mg/kg (3 mg/kg, 6 doses, days 0, 4, 8, 15, 22, and 29, IP)	↑Drp1 in mitochondria ↑p-Drp1^ser616^ ↓Mfn1 ↓Mfn2 ↓Opa1 ↑Mitochondrial volume density ↓Mitochondrial area	↓RCR ↑ROS ↑MMP depolarization ↑Swelling ↑Parkin ↑Beclin-1 ↑p62 ↑LC3II/I	↑MDA in serum and tissue ↑TNF-a ↑IL-6 ↑Bax ↑c-Caspase3 ↑Cyt c ↑TUNEL-positive cells	↓HR ↓LVESP ↓+dp/dt ↓SV ↓SBP ↓DBP ↓EF ↓FS ↓E/A ratio ↑LVEDP ↑-dp/dt ↑LF/HF ratio ↑NT-proBNP ↑cTn-I	DOX altered mitochondrial dynamics, mitochondrial dysfunction, autophagy and mitophagy activation, increased oxidative stress, inflammation, and apoptosis, leading to cardiac dysfunction.	[16]
Male Wistar rats	18 mg/kg (3 mg/kg, 6 doses, days 0, 4, 8, 15, 22, and 29, IP)	↑Drp1 in mitochondria ↑p-Drp1^ser616^ ↓Mfn1 ↓Mfn2	↓RCR ↑ROS ↑MMP depolarization ↑Swelling ↑PINK1 ↑Parkin ↑Beclin-1 ↑p62 ↑LC3II/I	↑MDA in serum and tissue ↑TNF-a ↑IL-6 ↑Bax ↑c-Caspase3 ↑TUNEL-positive cells	↓HR ↓SV ↓SBP ↓DBP ↓EF ↓FS ↓E/A ratio ↑LF/HF ratio ↑NT-proBNP ↑c-TnI ↓a7nAChR ↓M2AChR	DOX impaired both a7nAChR and M2AChR, leading to altered mitochondrial dynamics, mitochondrial dysfunction, autophagy and mitophagy activation, increased oxidative stress, inflammation, apoptosis, and cardiac dysfunction.	[15]
Male and female mixed FVB/C57BL/6 mice	18 mg/kg (6 mg/kg, every third day for a week, 14 days, IV)	↑Mfn1 ↑Fis1 ↑Mitochondrial number ↓Mitochondrial area	↓mtDNA ↑Nrf1 ↑PINK1 ↑Parkin	↓HO-1 ↑CD11b^+^	↓EF ↑EDD ↑ESD ↑LVEDD ↑LVESD ↓LVPWtd ↓LVPWts	DOX-induced dilated cardiomyopathy, cardiac cytoarchitectural derangement, infiltration of mononuclear phagocytes, dilation of the sarcoplasmic reticulum, mitochondrial fragmentation, and increased numbers of damaged mitochondria in autophagic vacuoles.	[50]
Male Wistar rats	18 mg/kg (3 mg/kg, 6 doses, days 0, 4, 8, 15, 22, and 29, IP)	↑Drp1 in mitochondria ↑p-Drp1^ser616^ ↓Mfn1 ↓Mfn2 ↓Opa1 ↑Mitochondrial volume density ↓Mitochondrial area	↓RCR ↑ROS ↑MMP depolarization ↑Swelling ↑Parkin ↑Beclin-1 ↑p62 ↑LC3II/I	↑MDA in serum and tissue ↑TNF-α ↑IL-6 ↑Bax ↑c-Caspase3 ↑Cyt c ↑TUNEL-positive cells	↓HR ↓LVESP ↓+dp/dt ↓SV ↓SBP ↓DBP ↓EF ↓FS ↓E/A ratio ↑LVEDP ↑-dp/dt ↑LF/HF ratio ↑NT-proBNP ↑cTn-I	DOX caused cardiac dysfunction by altering mitochondrial dynamics, dysfunction, autophagy, mitophagy, oxidative stress, inflammation, and apoptosis.	[13]
Male Wistar rats	18 mg/kg (3 mg/kg, 6 doses, days 0, 4, 8, 15, 22, and 29, IP)	↑Drp1 in mitochondria ↑p-Drp1^ser616^ ↓Mfn1 ↓Mfn2 ↓Opa1 ↑Mitochondrial volume density ↓Mitochondrial area	↓RCR ↑ROS ↑MMP depolarization ↑Swelling ↑Parkin ↑Beclin-1 ↑p62 ↑LC3II/I	↑MDA in serum and tissue ↑TNF-α ↑IL-6 ↑Bax ↑c-Caspase3 ↑Cyt c ↑TUNEL-positive cells	↓HR ↓LVESP ↓+dp/dt ↓SV ↓SBP ↓DBP ↓EF ↓FS ↓E/A ratio ↑LVEDP ↑-dp/dt ↑LF/HF ratio ↑NT-proBNP ↑cTn-I	DOX altered mitochondrial dynamics, mitochondrial dysfunction, autophagy and mitophagy activation, increased oxidative stress, inflammation, and apoptosis, leading to cardiac dysfunction.	[14]
Male C57BL/6 mice	20 mg/kg (5 mg/kg, at first, third, fifth and seventh day, IP)	↓Mfn2	↓PPAR-a ↓PGC1-a ↓Sirt3 ↑PERK ↑ATF4 ↑CHOP	↑LDH ↓SOD ↑MDA ↓GSH ↓CAT ↓Nrf2 ↑Keap1 ↓Bcl-2 ↑Bax ↑Caspase9 ↑Cyt c	↓EF ↓FS ↓E/A ratio ↑CKMB ↑BNP ↑c-TnT ↑Fibrosis	DOX induced cardiotoxicity by promoting mitochondrial dysfunction and ER stress by activating MFN2/PERK.	[46]
Male C57BL/6 mice	20 mg/kg (5 mg/kg, weekly for 4 consecutive weeks, IP)	↑Fragmented mitochondria ↑MIEF2	-	↓Foxo3a ↑TUNEL-positive cells	↑LVIDd ↓FS	Foxo3a is downregulated in the mouse heart in response to DOX, leading to increased mitochondrial fission and apoptosis.	[42]
Male C57BL/6J mice	20 mg/kg (5 mg/kg, weekly for 4 weeks, IP)	↓Drp1 ↔Mfn2 ↔Opa1	↔LC3II ↔p62 ↔Beclin1 ↔ATG7 ↔LAMP2 ↔p-AMPK ↑ULK1^ser757^ ↑p-mTOR ↑Parkin ↓PGC1-a ↑NRF1 ↑COX1 ↑COX2	↑4HNE ↑IL-1b ↑c-Caspase3 ↑TUNEL-positive cells	↑Abnormal morphology	DOX induced oxidative stress, apoptosis, and tissue damage without affecting autophagy or mitochondrial dynamics.	[51]
FVB/NJ mice	20 mg/kg (single dose, euthanized at day 3, IP)	↓Opa1 ↓Fis1 ↑Fragmented mitochondria	↓ATP ↑LC3II ↑P62 ↓Parkin	↑LDH ↑ROS	↑Fibrosis ↑CKMB	DOX induced the accumulation of fragmented mitochondria, cytoplasmic vacuolization, an increase in oxidative stress, and an increase in the thickness of the dysfunctional LV wall.	[52]
	16 mg/kg (4 mg/kg, weekly for 4 weeks, IP)	-	-	-	↓Systolic LVPW ↓Diastolic LVPW		
Female Balb/c mice	30 mg/kg (10 mg/kg, on days 2, 8 and 15, IP)	↑p-Drp1^ser616^	-	↑4-HNE ↑NQO1 ↑c-Caspase3	↓CO ↓EF ↓SV ↓FS	DOX induced cardiotoxicity by increasing the reactive oxygen species, mitochondrial damage, and apoptosis.	[45]

Abbreviations: 4HNE: 4-hydroxynonenal, or 4-hydroxy-2-nonenal; a7nAChR: alpha-7 nicotinic receptor; Akt: protein kinase B; ATG5: autophagy-related gene 5; ATP: adenosine triphosphate; Bax: Bcl-2-associated X protein; BNP: brain natriuretic peptide; BW: body weight; c-TnT: cardiac troponin T; CAT: catalase; CK: creatine kinase; CKMB: creatine kinase MB; CO: cardiac output; CS: mitochondrial citrate synthase; Cx43: connexin 43; CypD1: cyclophilin D; Cyt c: cytochrome c; Drp1: dynamin-related protein 1; E/A ratio: the ratio between E-wave and A-wave; EF: ejection fraction; Erk: extracellular signal-regulated kinase; Fis1: mitochondrial fission 1 protein; FoxO1: forkhead box O1; Foxo3: forkhead box O3; FS: fractional shortening; GSDMD-NT: gasdermin D-N terminal; GSH: glutathione; H2O2: hydrogen peroxide; HO-1: heme oxygenase 1; HR: heart rate; HW: heart weight; IC: intracoronary injection; IL: interleukin; IP: intraperitoneal injection; Keap1: Kelch-like ECH-associated protein 1; LC3: microtubule-associated protein 1 light chain 3; LDH: lactate dehydrogenase; LF/HF ratio: low-frequency to high-frequency ratio; LVDP: left ventricular developed pressure; LVEDD: left ventricular end-diastolic diameter; LVEF: left ventricular ejection fraction; LVESD: left ventricle end-systolic dimension; M2AchR: muscarinic acetylcholine receptor M2; MDA: malondialdehyde; Mfn1: mitofusin 1; Mfn2: mitofusin 2; MIEF2: mitochondrial elongation factor 2; MMP: mitochondrial membrane potential; NFkB: nuclear factor kappa-light-chain-enhancer of activated B cells; NLRP3: NLR family pyrin domain-containing 3; NOX: NADPH oxidases; NQO1: NAD(P)H: quinone oxidoreductase 1; Nrf1: nuclear respiratory factor 1; Nrf2: nuclear respiratory factor 2; NT-proBNP: N-terminal pro B-type natriuretic peptide; OGG1: 8-oxoguanine DNA glycosylase-1; Opa1: optic atrophy type 1; OXPPHOS: oxidative phosphorylation; p-Drp1ser616: phosphorylation of dynamin-related protein 1 at serine 616; p-Drp1 ser637: phosphorylation of dynamin-related protein 1 at serine 637; p-PDH: pyruvate dehydrogenase; PARP: poly (ADP-ribose) polymerase; PGC1-α: peroxisome proliferator-activated receptor gamma coactivator 1-alpha; RCR: respiratory control ratio; ROS: reactive oxygen species; SIRT1: sirtuin 1; Sirt3: sirtuin 3; SOD: superoxide dismutase; TLR-4: Toll-like receptor 4; TNF-α: tumor necrosis factor alpha.

## Data Availability

Not applicable.
